# Estimated prevalence of obstructive sleep apnea by occupation and industry in England: a descriptive study

**DOI:** 10.1093/sleepadvances/zpae069

**Published:** 2024-09-18

**Authors:** Ryohei Kinoshita, Jennifer K Quint, Constantinos Kallis, Michael I Polkey

**Affiliations:** School of Public Health, Faculty of Medicine, Imperial College London, London, UK; School of Public Health, Faculty of Medicine, Imperial College London, London, UK; School of Public Health, Faculty of Medicine, Imperial College London, London, UK; Royal Brompton Hospital, Guys and St Thomas NHS Foundation Trust, London, UK; National Heart and Lung Institute, Imperial College London, London, UK

**Keywords:** obstructive sleep apnea, prevalence, occupation, industry, multiple imputation

## Abstract

**Study Objectives:**

Obstructive sleep apnea (OSA) can induce excessive sleepiness, causing work-related injuries and low productivity. Most individuals with OSA in the United Kingdom are undiagnosed, and thus, theoretically, workplace screening, might by identifying these individuals improve both their individual health and overall productivity. However, the prevalence of OSA in different workplaces is unclear. This study aimed to estimate the prevalence of OSA by industries and occupations in England.

**Methods:**

The Health Survey for England 2019 dataset was combined with Sleep Heart Health Study dataset. We applied multiple imputation for the combined dataset to estimate OSA in the English population aged 40–64. We estimated the pooled prevalence of OSA by both industry and occupation by separating samples by Standard Industry Classification and Standard Occupation Classification.

**Results:**

The overall OSA prevalence estimated by imputation for ages 40–64 was 17.8% (95% CI = 15.9% to 19.9%). Separating those samples into industrial/occupational groups, the estimated prevalence of OSA varied widely by industry/occupation. Descriptive analysis revealed that the estimated prevalence of OSA was relatively higher in the Accommodation and food, Public administration and defence; compulsory social security, Construction industries, and Protective service occupations, health and social care associate professionals, and skilled construction and building trades occupations.

**Conclusions:**

In England in 2019, Accommodation and food, Public administration and defence; compulsory social security, Construction industries, and Protective service occupations, health and social care associate professionals, and skilled construction and building trades occupations showed a relatively higher prevalence of OSA indicating that they may be target populations for workplace screening.

**Statement of Significance**

Obstructive sleep apnea (OSA) is increasing in prevalence, but most patients are undiagnosed. Recently, low-cost technologies for OSA screening have emerged. It would be logical to start screening programs in those occupations/industries with the highest prevalence of OSA, but these are currently unknown. In this paper, we estimated the prevalence of OSA by multiple imputation methods and showed that in England in 2019 this ranged widely both by occupations and industries. The prevalence of OSA was higher in the Accommodation and food industry, the Public administration and defence; security industry, and the Protective service occupations, the health and social care associate professionals, and the construction industry and occupation. Thus, these sectors could be potential target populations for OSA screening in England.

Obstructive sleep apnea (OSA) is a disorder characterized by episodes of airway collapse, and consequently absent or reduced airflow during sleep. OSA is estimated to affect 425 million individuals globally [1]. Because of sleep fragmentation, OSA may cause excessive sleepiness, and thus be associated with occupational injuries (e.g. motor vehicle collisions) [2–4] and low labor productivity (e.g. presenteeism) [5, 6]. Continuous positive airway pressure (CPAP) reduces daytime sleepiness in people with OSA [7–9]. Nevertheless, the majority, up to 85%, of individuals with moderate to severe OSA, are undiagnosed [10–12].

Screening has been discussed as one of the solutions to improve the delivery of CPAP to people with undiagnosed OSA [13] and has become more feasible with the advent of lower-cost technologies such as mandibular movement monitoring [14]. Once individuals are identified, CPAP has been shown to improve personal health, and in addition, some evidence is available that CPAP reduces occupational injuries or improves productivity [7–9]. In one major trucking firm in the United States, screening and treatment for OSA reduced car crashes among truck drivers [15]. However, OSA screening has not been recommended globally due to a lack of evidence confirming its value [13, 16].

The effectiveness of screening depends in part on the prevalence of the screened condition in the target population; thus, knowledge of disease prevalence would inform which industries or occupations would be most fruitful to screen. In Sweden, the risk of OSA has been reported to vary by occupation [17], however, there are no data regarding the prevalence of OSA by occupations/industries for any other country including the United Kingdom. The United Kingdom comprises 4 constituent nations of which England is substantially the largest; approximately 85% of UK residents live in England, with the remainder in Scotland, Wales, and Northern Ireland. Here, we aimed to estimate the prevalence of OSA by industries and occupations in England, with the goal of identifying those industries and occupations that might be attractive for screening; data collection is devolved to the home nations so we could not include the 3 other nations of the United Kingdom.

We first needed to estimate OSA outcome data to estimate the prevalence of OSA in the general population in England. However, because there is no publicly available data on OSA outcome in the general population in the United Kingdom, we combined England’s population dataset with a US community cohort dataset containing OSA outcome data, then applied multiple imputation to the combined dataset to estimate the OSA outcome data in England’s population samples. Then, we separated England’s population into subgroups of industries or occupations and estimated the prevalence of OSA by industries. Subsequently, we undertook descriptive analysis to identify which industries have a relatively higher prevalence of OSA than other industries.

## Methods

### Study design

We used the Sleep Heart Health Study Visit 1 dataset (SHHS) for estimating OSA data in England’s population data. Health Survey for England 2019 dataset (HSE2019) was used as England’s population data. For process validation of multiple imputation, we used Wisconsin Sleep Cohort Visit 1 as a test dataset. We identified predictors for OSA from those datasets and harmonized predictors across three datasets. We combined HSE2019 with SHHS to estimate OSA data in HSE2019 by multiple imputation. Then, we estimated the prevalence of OSA in HSE2019 by industries and occupation. To validate the multiple imputation process, we applied the same process to estimate the prevalence of OSA in WSC and compared it with the prevalence based on the observation in WSC. Then, we did a descriptive analysis for differences in the estimated prevalence by industries and occupations. The study design diagram is shown in Supplementary Material S1. Study methods and results followed the STROBE Statement checklist for cross-sectional studies [18].

### Data sources and population

#### Sleep Heart Health Study.

We used the SHHS dataset for multiple imputation for OSA data in HSE2019 dataset. The SHHS Visit 1 [19] was a large cohort study that assessed the relationship between phenotype and OSA in individuals aged ≥ 40 [20, 21]. The SHHS dataset contains both OSA outcomes and OSA risk factors. Although the data were collected from an American population, we adopted the SHHS dataset since there are no accessible population datasets containing both OSA outcomes and OSA risk factors in England. The sample size of SHHS Visit 1 was 5804.

#### Health Survey for England.

We used the HSE2019 dataset to estimate the prevalence of OSA in England. HSE2019 was the latest publicly available dataset in England. The HSE is a series of annual national health surveys from a representative population across England [22]. Participants were randomly selected across ages and regions in England. Data were collected through Computer-Assisted Personal Interviewing [22]. The HSE2019 [23] dataset contains data of risk factors for OSA and classification of occupation and industry, but does not contain outcome data of OSA. The sample size of the HSE2019 dataset was 21 791.

#### Wisconsin Sleep Cohort.

We used the Wisconsin Sleep Cohort (WSC) dataset for validation of multiple imputation model. WSC is a longitudinal study assessing sleep disorders. The WSC dataset contains both OSA outcomes and OSA risk factors in general population [21, 24]. The setting is Wisconsin state in the United States. We selected Visit 1 data from five visits of the cohort study. The sample size of Visit 1 of the WSC dataset is 1123.

As the SHHS dataset did not include participants aged < 40 [20, 21], we excluded participants aged < 40 from the WSC and HSE2019 datasets. Similarly, since we were concerned with occupational screening, we excluded participants aged ≥ 65, the conventional retirement age in England, from all datasets.

### Variables

#### Outcome.

The outcome variable was an Apnea-Hypopnea Index (AHI) ≥ 15 events/hour, considered to constitute a diagnosis of OSA. Apnea was a state with no oxygen desaturation threshold used and with or without arousal during sleep tests. The definition of hypopnea was a state with greater than 30% or discernible flow reduction and at least 4% oxygen desaturation and with arousal during sleep tests [25]. The present study focused on AHI ≥ 15 because, in terms of a beneficial screening program, moderate to severe OSA is more likely to need resources for treatment than mild OSA, such as CPAP [26]. We converted continuous AHI data into a binary of AHI ≥ 15 since the primary focus of our study was the diagnosis itself as opposed to its severity.

#### Predictors.

We extracted candidate predictors from all datasets based on the literature and experts’ opinions, including age, sex, ethnicity, obesity, genetic factor, craniofacial shape, neck circumference, socioeconomic status, smoking, alcohol, sodium intake, menopause, fluid retention/edema, adenotonsillar hypertrophy, GERD, analgesic use, hypertension, cardiovascular disease, diabetes, hyperlipaemia, chronic kidney disease, and hypothyroidism (Supplementary Material S2) [27–43]. Excluding unavailable variables across three datasets, we consequently included age, sex, ethnicity, obesity, smoking, alcohol, hypertension, diabetes, and hyperlipaemia as candidate predictors. For variable selection, we drew a directed acyclic graph (DAG) by DAGitty program version 3.0 (Supplementary Material S2) [44, 45]. The DAG suggested adjusting age, ethnicity, and physical activity to reduce biasing paths. However, physical activity data was unavailable in the SHHS dataset. The DAG also suggested that adjusting hyperlipaemia introduces more biasing paths. Therefore, we considered incorporating age and ethnicity in the predictors and excluding hyperlipaemia from the predictors. We used BMI as a continuous variable. On the other hand, we used categorical data for other variables because one of the datasets only contained categorical data, which thus not able to be harmonized across all datasets. Descriptions about handling each data are in Supplementary Material S3.

#### Exposure: industry and occupation.

We used the Standard Industry Classification (*SIC*) and the Standard Occupation Classification (SOC) to estimate the prevalence of OSA by occupations and industries. The *SIC* represents classifications of economic activities, and the SOC of jobs, and both comprehensively cover all occupations or industries in England [46, 47]. We shortened the group names of SIC2007 and SOC2010 for our study (Supplementary Material S4).

#### Survey weights.

We used survey weights through the analysis (Supplementary Material S3). We used individual weight data provided by HSE. As the WSC weighted high risk of people with sleep disorder with 1.5 and low risk of them with 1, we assigned 1.5 for high risk and 1 for low risk. The SHHS combined six separate studies with complex sampling designs. It was difficult to estimate weight values for individuals in SHHS, so we assigned 1 for participants of SHHS.

### Statistical methods

#### Multiple imputation for missing values.

We combined the HSE2019 dataset with the SHHS dataset to apply multiple imputation for missing data including OSA outcome data in the combined dataset. To assess if the integration of two different datasets was viable, we compared observed data in SHHS and HSE2019. Specifically, we compared proportions and missingness for categorical variables, and mean, median, histogram, and missingness for continuous variables, assessing if those two datasets have substantial differences. Then, to assess whether a missing at random (MAR) assumption for multiple imputation was reasonable, we explored the patterns of missingness in the combined dataset. Specifically, we observed the number of missing data and identified major causes from the dataset and protocol document of the dataset [22], assessing if the missingness depended on their values or not.

We used a *mice* command [48] to apply multiple imputation for missing values with 10 imputations based on 20 iterations. We included all predictors defined in the section of predictors for multiple imputation models, applying predictive mean matching for numerical data, logistic regression for binary data, and proportional odds model for categorical with more than two categories. Survey weights were incorporated in multiple imputation models described above.

Following the implementation of multiple imputation, we fitted a logistic regression model on ten multiple imputed datasets using binary OSA data as an outcome and all other variables used in the multiple imputation as predictors for the logistic regression model. The survey weights were incorporated into the model. To obtain a single set of estimates of odds ratio, 95% CI, and *p*-value of the logistic regression model, we pooled these estimates from ten multiply imputed models by using *pool* command [49] in R package *mice* after. For descriptive analysis of participants’ characteristics after multiple imputation, we calculated the pooled single proportions and CIs of each categorical predictor by a command *pool_prop_wilson* in HSE2019 [50]. For continuous predictors, we calculated the mean and 95% CI of the predictor by null-linear regression models setting the predictor as a dependent variable and pooling intercept coefficients from ten imputed datasets using the *pool* command. We compared the proportions or mean of participants with OSA and without OSA, using *pool_D2* [51] for categorical and *pool_t_test* [52] for continuous variables. We compared the proportions or mean of each covariate before and after the multiple imputation, using an observed dataset and a first-completed dataset extracted from ten datasets.

To validate the multiple imputation process using the SHHS dataset for estimating the prevalence in other datasets, we estimated the prevalence of OSA in the WSC dataset by multiple imputation, assessing if the estimated prevalence approximates the observed prevalence in the WSC dataset. We combined WSC dataset with SHHS dataset, then applied the same imputation process to impute OSA data of WSC samples, estimating the pooled prevalence of OSA in WSC data. Then, we compared the actual (observed) prevalence of OSA and the corresponding imputed prevalence using estimates from the imputation model. Survey weights were used throughout the analysis.

#### Prevalence of OSA estimated by using multiple imputation.

Using HSE2019 dataset observations from multiple imputed datasets, we pooled OSA proportions to obtain the estimated prevalence of OSA in the whole of England. We also estimated 95% CIs of the prevalence of OSA using the Wilson method [50] across multiple imputed datasets.

We divided samples into *SIC* and SOC subgroups and then estimated the pooled proportions of OSA and Wilson CIs as the estimated prevalence for all industries and occupations separately. For descriptive analysis, we plotted the estimated prevalence and 95% CIs from largest to smallest by industry and occupation, identifying industries or occupations with a relatively higher prevalence.

We also estimated pooled differences in proportions of OSA between industries and occupations across multiple imputed datasets in England’s population. We estimated Newcombe-Wilson (NW) CIs of the pooled differences in proportions of OSA between industries and occupations [53]. We used R version 4.4.1 (Vienna, Austria) for all analyses.

### Ethical approval

This study did not require ethical approval as we were using publicly available datasets with no patient-identifiable data.

## Results

### Study population

The number of participants aged 40–64 was 3122 in SHHS and 3437 HSE2019, respectively (Supplementary Material S5). Age was skewed to the lower side in HSE2019 compared to SHHS. HSE2019 included more self-reported drinkers of alcohol than SHHS (2061, 60.2% vs 1404, 50.4%) aged 40–64. Missingness was high in BMI among people aged 40–64 in HSE2019 (597). Other variables did not show substantial differences between SHHS and HSE2019, so we combined SHHS and HSE2019. Although the missingness in BMI from HSE2019 was large, we concluded that MAR in the combined dataset was reasonable based on major reasons for the missingness provided by HSE2019 and the survey protocol (Supplementary Material S5).

### Multiple imputation

In the multiple imputation model, the predictor terms with *p*-values below 0.05 included age 60–64, male, BMI, and hypertension. No subgroups of *SIC* or SOC showed *p*-values below 0.05 (Supplementary Material S6).

The validation model showed similar coefficients to the imputation model above (Supplementary Material S6). The pooled proportion of OSA in WSC test data was 16.1% (95% CI = 12.1% to 21.0%). The weighted prevalence of OSA in the observed data (19.5%) was within this CI.

The distribution of imputed data did not change significantly compared to observed data only (before multiple imputation; Table 1).

**Table 1.** Demographic and Anthropometric Characteristics of the Population in the HSE2019 Before and After Multiple Imputation (age 40–64)

Characteristic	Observed, *N* = 3437, *n* (%)^1^	Completed, *N* = 3437, *n* (%)^1^
Age		
40–59	2781 (80.9%)	2781 (80.9%)
60–64	656 (19.1%)	656 (19.1%)
NA	0	0
Sex		
Female	1879 (54.7%)	1879 (54.7%)
Male	1558 (45.3%)	1558 (45.3%)
NA	0	0
Ethnicity		
White	2927 (85.5%)	2939 (85.5%)
Non-white	497 (14.5%)	498 (14.5%)
NA	13	0
BMI		
Mean (SD)	28.50 (5.7)	28.51 (5.7)
NA	597	0
Smoking		
Never	1624 (47.4%)	1628 (47.4%)
Former	1241 (36.2%)	1248 (36.3%)
Current	559 (16.3%)	561 (16.3%)
NA	13	0
Alcohol		
Non-drinker	1363 (39.8%)	1371 (39.9%)
Drinker	2061 (60.2%)	2066 (60.1%)
NA	13	0
Hypertension		
Non-hypertension	2363 (68.8%)	2363 (68.8%)
Hypertension	1074 (31.2%)	1074 (31.2%)
NA	0	0
Diabetes		
Non-diabetes	3106 (90.4%)	3109 (90.5%)
Diabetes	328 (9.6%)	328 (9.5%)
NA	3	0
OSA		
Non-OSA or Mild OSA	0 (NA%)	2816 (81.9%)
Moderate to severe OSA	0 (NA%)	621 (18.1%)
NA	3437	0
SIC2007		
(ABDE) Agriculture, Mining, Energy, Utilities and Waste	79 (2.4%)	83 (2.4%)
(C) Manufacturing	343 (10.3%)	357 (10.4%)
(F) Construction	256 (7.7%)	267 (7.8%)
(G) Retail and repair	399 (12.0%)	403 (11.7%)
(H) Transportation and storage	246 (7.4%)	252 (7.3%)
(I) Accommodation and food	121 (3.6%)	127 (3.7%)
(J) Information and communication	108 (3.3%)	109 (3.2%)
(K) Financial activities	92 (2.8%)	95 (2.8%)
(L) Real estate	23 (0.7%)	24 (0.7%)
(M) Professional services	210 (6.3%)	221 (6.4%)
(N) Administrative support	193 (5.8%)	197 (5.7%)
(O) Public services	201 (6.0%)	209 (6.1%)
(P) Education	388 (11.7%)	399 (11.6%)
(Q) Healthcare and social work	507 (15.3%)	526 (15.3%)
(R) Arts and entertainment	65 (2.0%)	70 (2.0%)
(Others) Others	92 (2.8%)	98 (2.9%)
NA	114	0
SOC2010		
(11) Corporate management	235 (7.1%)	235 (6.8%)
(12) Other management	117 (3.5%)	117 (3.4%)
(21) Science and tech professionals	142 (4.3%)	146 (4.2%)
(22) Health professionals	144 (4.3%)	147 (4.3%)
(23) Teaching professionals	162 (4.9%)	173 (5.0%)
(24) Business and media pros	160 (4.8%)	174 (5.1%)
(31) Tech associate pros	41 (1.2%)	42 (1.2%)
(32) Health and social care pros	46 (1.4%)	46 (1.3%)
(33) Protective services	38 (1.1%)	38 (1.1%)
(34) Culture and sports jobs	63 (1.9%)	63 (1.8%)
(35) Business pros	221 (6.7%)	224 (6.5%)
(41) Administrative jobs	309 (9.3%)	315 (9.2%)
(42) Secretarial jobs	99 (3.0%)	102 (3.0%)
(51) Agricultural trades	43 (1.3%)	45 (1.3%)
(52) Metal and electrical trades	108 (3.3%)	108 (3.1%)
(53) Construction trades	119 (3.6%)	119 (3.5%)
(54) Crafts and printing	71 (2.1%)	71 (2.1%)
(61) Caring services	280 (8.4%)	291 (8.5%)
(62) Leisure and travel services	73 (2.2%)	78 (2.3%)
(71) Sales jobs	174 (5.2%)	188 (5.5%)
(72) Customer service	37 (1.1%)	37 (1.1%)
(81) Machine operatives	134 (4.0%)	152 (4.4%)
(82) Transport and drivers	141 (4.2%)	149 (4.3%)
(91) Elementary manual trades	49 (1.5%)	53 (1.5%)
(92) Elementary administration	317 (9.5%)	324 (9.4%)
NA	114	0

^1^A completed dataset was extracted from a 10-multiple imputed dataset. Abbreviations: OSA, obstructive sleep apnea, Health Survey for England; *SIC*, Standard Industry Classification; SOC: Standard Occupation Classification.

### Estimated prevalence

The estimated prevalence of OSA was 17.8% (95% CI = 15.9% to 19.9%) among people aged 40–64 in England in 2019. Summary characteristics of participants with OSA and without OSA after multiple imputation is shown in Supplementary Material S7. Comparing participants predicted to have OSA and others, all covariates were not different.

**Figure 1.**
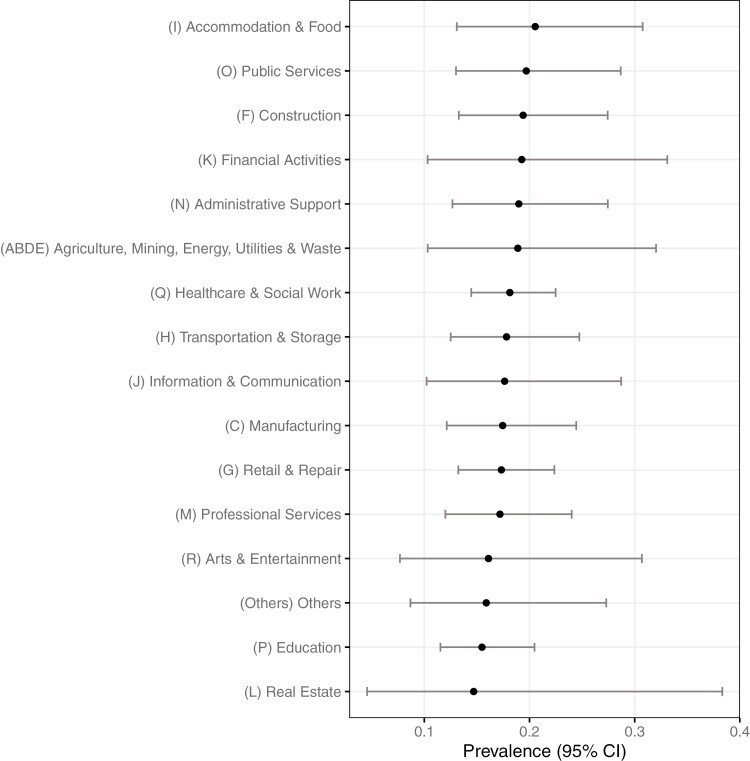
Estimated prevalence of OSA (%) by industries in England in 2019 (age 40–64). Industries were classified by SIC2007. Lines are 95% CI. Abbreviations: OSA, obstructive sleep apnea; *SIC*, Standard Industry Classification.

The estimated prevalence of OSA and their CIs varied by industries in England in 2019 (Figure 1; Supplementary Material S8). The estimated prevalence of OSA in the Accommodation and food service activities (*SIC*-I), the Public administration and defence; compulsory social security (*SIC*-O), and the Construction (*SIC*-F) were top three among all industries (Figure 1). The 95% CIs of differences in OSA prevalence was overlapping zero between all industry groups (Supplementary Material S8).

**Figure 2.**
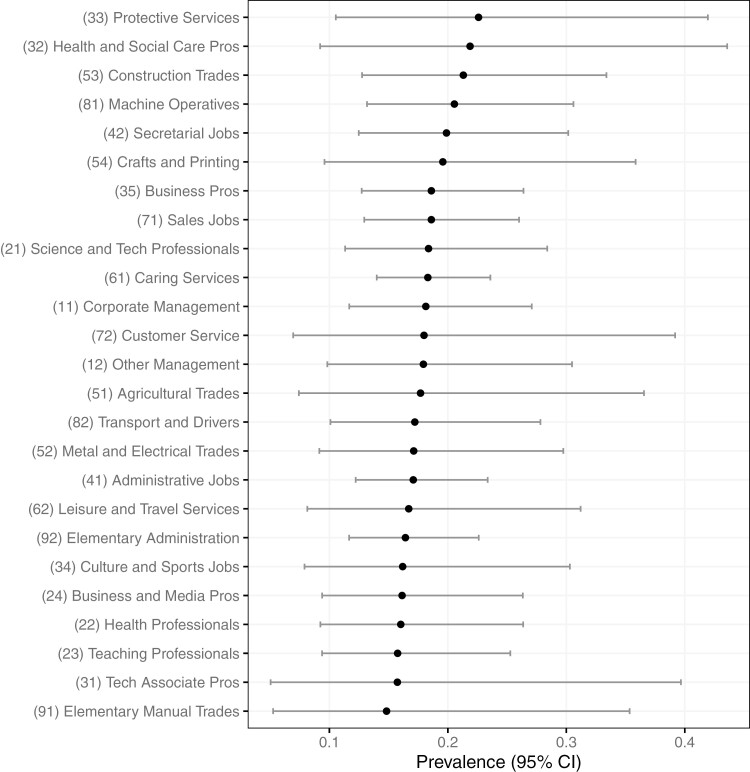
Estimated prevalence of OSA (%) by occupations in England in 2019 (age 40–64). Occupations were classified by SOC2010. Lines are 95% CI. Abbreviations: OSA, obstructive sleep apnea; SOC, Standard Occupation Classification.

The estimated prevalence of OSA also ranged widely by occupation (Figure 2; Supplementary Material S9), showing that protective services (SOC-33), health and social care pros (SOC-32), and construction trades (SOC-53) were the top three occupations. The 95% CIs of differences in the estimated prevalence of OSA were not seen between all occupation groups (Supplementary Material S9).

## Discussion

As expected, the estimated prevalence of OSA is high amongst 40- to 64-year-olds in the United Kingdom. Although there were wide CIs that often overlapped when considering different industrial sectors or different occupations, the data nevertheless provide a hierarchy of estimated prevalence in different sectors and trades. This structure could be used in conjunction with consideration of the possible consequences of a sleep-related accident in those workplaces to provide a logical basis to choose where to screen for OSA.

### Comparison with other studies

There are few studies in this area but our data are consistent with prior studies. A Swedish study [17] reported cooks as occupations at high risk of OSA. Our study also showed a relatively higher estimated prevalence of OSA in the accommodation and food service activities (*SIC*-I), again consistent with the Swedish study.

In the United States, the prevalence of OSA in firefighters was 28.4% [54]. In our study, the security industry (*SIC*-O) and the protective service occupation (SOC-33) showed a relatively higher prevalence of OSA, including safety-sensitive occupations, such as police officers, firefighters, and traffic wardens. The associations in these industries and occupations may therefore be generalizable in other countries.

Additionally, our study also identified construction (*SIC*-F and SOC-53) as an area with a high prevalence of OSA. Although there are no comprehensive studies about the prevalence of OSA in the construction industry in the United Kingdom, a cross-sectional study in Iran reported that 37.2% of workers in a construction company had OSA-associated symptoms [55].

Health and social care associate professionals (SOC-32) also had a greater prevalence of OSA in our study. A cross-sectional study analyzing HSE 2008–2012 revealed that the prevalence of obesity in unregistered care workers (31.9%, 95% CI = 28.4% to 35.3%) was higher than in non-health-related occupations (23.5%, 95% CI = 22.9% to 24.1%) [56]. Considering the high prevalence of obesity in the Health and social care associate professionals, it is plausible that OSA is prevalent in this occupation in England.

### Implications for clinicians and policymakers

Although it is established that drivers with OSA have an increased risk for occupational injuries, the relationship between OSA and injuries in other occupations is largely unknown. The British Lung Foundation reported that 22% of people with OSA at sleep clinics had a job that required regular driving [57]. Existing DVLA and NICE guidelines recommended prioritizing those in safety-sensitive occupations including drivers of heavy goods vehicles, buses, and trains [26, 58]. However, our findings suggest a high prevalence of OSA in several other occupations, including the security industry (*SIC*-O), the protective service occupation (SOC-33), the construction industry (*SIC*-F), and the skilled construction and building trades (SOC-53). A study using a US national sample of firefighters revealed a high rate of falling asleep during driving and motor vehicle crashes [54]. In terms of construction industry/occupation, a case–control study among construction workers in France showed that sleep disorder was a significant factor associated with falls to a lower level [59]. Therefore, sleep-related accidents in these industries/occupations could have serious consequences, and who we believe should also now be prioritized by national guidelines and policymakers in the United Kingdom.

### Future research

Future studies should directly test our imputation model by evaluating it against a direct screening measurement, with the aim of testing its accuracy. If physiological data are supportive, we recommend a trial of screening for (and treatment of) OSA in one of the industries with a high estimated prevalence of OSA identified here. The first of these studies could be nested within the second if desired. Outcomes should include both those relevant to the individual (e.g. sleepiness, quality of life) and societal (e.g. sleep-related accidents, productivity, and levels of sickness).

Should such an approach prove feasible, further work would be required to expand the model to confirm its functionality in those of non-Caucasian ancestry, those aged < 40, and those in occupations that with too few employees to be assessed in the current study.

Finally, we recognize that our data only estimated the prevalence of OSA, not the symptom impacts experienced by the individual, and it is well recognized that not all patients with OSA experience unwanted sleepiness; since sleepiness data were contained within SHHS and WSC a similar modeling approach could be used to estimate the prevalence of sleepiness by occupation and see where that dataset overlapped with the current one.

### Limitations

We used polysomnographically determined OSA data from a US cohort study for our imputation model to estimate OSA data in the UK population since there was no dataset containing OSA data in the UK population. However, the most of participants in both datasets were Caucasian, which means that the ethnicity of participants in the source dataset was similar to US and UK datasets in this study. Thus, the source dataset was viable for the imputation process, however our data cannot be uncritically translated to the majority non-Caucasian populations.

We used AHI based on the hypopnea definition with at least 4% oxygen desaturation rather than 3%. The American Academy of Sleep Medicine (AASM) 2012 update of the 2007 Manual recommended a 3% threshold for hypopnea [25], but our study could not use the 3% threshold since only data with a 4% threshold were available in the WSC dataset. Using a 4% threshold may, therefore, have underestimated the prevalence of OSA in England’s population. However, to identify appropriate industries for workplace screening, which was the aim of our study, using the 4% threshold may have advantages because it would identify population at higher risk of OSA than people identified with the 3% threshold. Moreover, the choice of desaturation threshold does not influence the relative risk between different industries and occupations.

In our data, we had few patients of far east Asian heritage. OSA is more prevalent at any given BMI in those of Far East Asian heritage than those of Caucasian heritage [60, 61]. Not considering Asian ethnicity might have tended to result in an underestimation of the OSA prevalence. However, the impact of low sample size of Asian participants would be small since the proportion of non-white participants was small, but it does mean our data may need recalibrating before applying to countries with a majority Asian population.

We could not consider cardiovascular disease as a predictor because of a lack of consistent data, with the exception of hypertension, across datasets. Had those data been present, we might have obtained narrower CIs for the prevalence of OSA, given the known link with cardiovascular disease. More generally we note that there was data in SHHS and WSC, particularly physical activity, which was not measured in HSE; if these data were collected in future HSE sampling rounds it might be possible to repeat the analyses and narrow the CIs. Similarly, any factors that influence OSA prevalence that differ among occupations or industries would also influence the prevalence estimate; as noted above an example of this might include an occupation that employed a disproportionate number of people of Far East Asian extraction.

The large proportion of missingness in BMI in the UK population dataset may have increased the uncertainty of the estimated prevalence of OSA by the multiple imputation in England’s dataset, reflected in the wide CIs in Figures 1 and 2. According to the pooled coefficient of multiple imputation model, the BMI seemed to be strongly related to OSA, but England’s dataset contained a large missingness in BMI. In general, the prevalence of obesity is higher in the United States than the United Kingdom [62], so the mean of BMI in HSE2019 participants may have been overestimated due to the large missingness, pushing the estimated prevalence higher. However, as the aim of our study was to identify industries with a relatively higher prevalence of OSA, the estimated prevalence of OSA by industries was interpretable.

Although we incorporated survey weights through analysis, we could not consider the sampling design of SHHS. The model was generated using SHHS data which in turn comprised six component studies all of which started with slightly differing aims. Many studies have used the SHHS dataset for secondary data analysis of OSA, but those studies did not incorporate survey weights. It could be argued that we should use complex survey weights of SHHS to try to improve the accuracy of our modeling.

## Conclusions

In England, in 2019, multiple imputation revealed a wide range of estimated prevalence of OSA whether categorized by industries or occupation. Since low-cost screening approaches are now available we suggest these data be used for a trial of screening for OSA in a high-risk population.

## Supplementary Material

zpae069_suppl_Supplementary_Materials_1-9

## Data Availability

The datasets of the Sleep Heart Health Study (https://doi.org/10.25822/ghy8-ks59) and Wisconsin Sleep Cohort (https://doi.org/10.25822/js0k-yh52) providing OSA outcome data and other health data used in this study are publicly available in the National Sleep Research Resources. The dataset of the Health Survey for England (https://doi.org/10.5255/UKDA-SN-8860-1) providing health data in general population was publicly available in the UK Data Service, being responsible by the NHS Digital. All datasets require providers’ approvals.
