# Supplementary material for: Estimated prevalence of obstructive sleep apnea by occupation and industry in England: a descriptive study
Source: Sleep Adv. 2024 Sep 18;5(1):zpae069. doi: 10.1093/sleepadvances/zpae069 (PMC11452655; doi:10.1093/sleepadvances/zpae069)
Supplement: zpae069_suppl_Supplementary_Materials_1-9 [file zpae069_suppl_supplementary_materials_1-9.docx]

Supplementary materials

Estimated prevalence of obstructive sleep apnoea by occupation and industry in England: a descriptive study

Ryohei Kinoshita^1^, Jennifer K Quint^1^, Constantinos Kallis^1^, Michael I Polkey^2 3^

1: School of Public Health, Faculty of Medicine, Imperial College London, UK.

2: Royal Brompton Hospital, Guys and St Thomas NHS Foundation Trust, Sydney Street, London SW3 6NP

3: Imperial College London, UK

Full address of corresponding author: Ryohei Kinoshita, Level 2, Faculty Building, South Kensington Campus, London SW7 2AZ, UK

Email address of corresponding author: ryohei.kinoshita21@alumni.imperial.ac.uk

Table of Contents

[Supplementary material 1: Study design 3](#_Toc173349963)

[Supplementary material 2: Predictor selection 4](#_Toc173349964)

[Supplementary material 3: Data preparation 10](#_Toc173349965)

[Supplementary material 4: Industries and Occupations 13](#_Toc173349966)

[Supplementary material 5: Characteristics of study participants 15](#_Toc173349967)

[Supplementary material 6: Multiple imputation model 18](#_Toc173349968)

[Supplementary material 7: Pooled summary characteristics of study participants 21](#_Toc173349969)

[Supplementary material 8: Estimated prevalence of OSA by industries in England 23](#_Toc173349970)

[Supplementary material 9: Estimated prevalence of OSA by occupations in England 25](#_Toc173349971)

[References 28](#_Toc173349972)

## Supplementary material 1: Study design


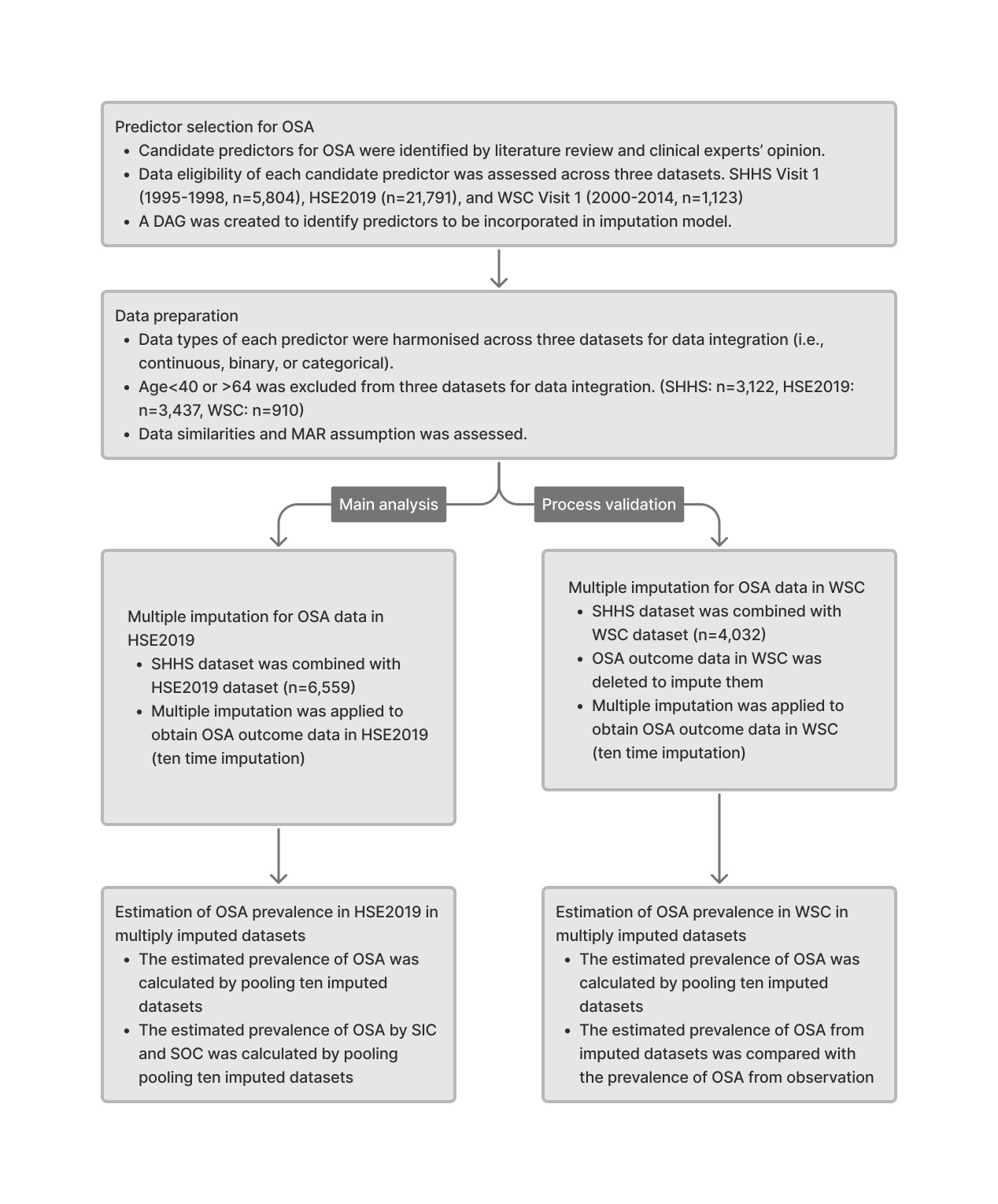


Figure S1. Study design diagram

## Supplementary material 2: Predictor selection

To select predictor variables for the multiple imputation model, we extracted risk factors for OSA from three datasets. Then, we checked their data availabilities across multiple datasets. We identified risk factors for OSA from literature review from 2010 onwards, which were mainly systematic reviews and some recent papers, adding risk factors according to clinical experts’ opinions (Table S1). Subsequently, we assessed data heterogeneity to exclude variables that were not included in all datasets consistently.

Based on the literature review and experts’ opinions, the candidate predictors included age,[1-7] sex,[2-4,6,8] ethnicity,[3,4,6,9,10] obesity,[1-5,7-11] smoking,[2,3,5,8] alcohol,[3,4,7] hypertension,[4,7,8,12-15] diabetes,[4,8,16,17] hyperlipaemia,[8] and cardiovascular disease.[4,12] Although the SHHS datasets contained comprehensive disease history data related to cardiovascular disease (e.g. myocardial infarction, congestive heart failure), the HSE2019 and WSC datasets only contained simple data for cardiovascular disease (Table S1). Thus, we excluded cardiovascular disease from candidate predictors. As a result, candidate predictors included age, sex, ethnicity, obesity, smoking, alcohol, hypertension, diabetes, and hyperlipaemia.

Table S1. OSA risk factors, relevant data (data type), and assessment for data harmonisation across all datasets

| **Risk factors** | **Relevant data** | | | **Assessment for data harmonisation** | | |
| --- | --- | --- | --- | --- | --- | --- |
|  | **SHHS[18]** | **HSE2019[19]** | **WSC[20]** | **Availability** | **Heterogeneity** | **Eligibility** |
| Age[1-7] | Age (continuous) | Age (categorical) | Age (continuous) | Available | Not heterogeneous | Eligible |
| Sex[2-4,6,8] | Gender (binary) | Sex (binary) | Sex (binary) | Available | Not heterogeneous | Eligible |
| Ethnicity[3,4,6,9,10] | Race (categorical): White/Black/Others | Ethnicity (categorical): White/Black/Asian/Mixed/multiple ethnic background/Others | Race (categorical): Asian/Black/Hispanic/Native American/White | Available | Not heterogeneous | Eligible |
| Obesity[1-5,7-11] | BMI (continuous) | BMI (continuous) | BMI (continuous) | Available | Not heterogeneous | Eligible |
| Genetic factor[10,14] | Not available | Not available | Not available | Not available | Not assessed | Not eligible |
| Craniofacial shape[3,6,9,10] | Not available | Not available | Not available | Not available | Not assessed | Not eligible |
| Neck circumference[3,5] | Neck circumference in centimetres (continuous) | Not available | Not available | Not available | Not assessed | Not eligible |
| Socioeconomic status[3] | Not available | Not available | Not available | Not available | Not assessed | Not eligible |
| Smoking[2,3,5,8] | Smoking Status (categorical): Never/Current/Former | 1: Ever smoked cigarette/cigar/pipe (categorical)  2: Whether smoke cigarettes nowadays (categorical)  3: Whether smoke cigar nowadays (categorical)  4: Whether smoke pipe nowadays (categorical) | 1: Ever smoke (binary)  2: Current smoking (binary) | Available | Not heterogeneous | Eligible |
| Alcohol[3,4,7] | Usual Alcohol Intake per day (continuous) | 1. Women number of alcohol units (categorical): None/Up to and including 3 units/Greater than 3 and less than or equal to 6 units/Greater than 6 units  2. Men number of units (categorical): None/<=4 units/day (men), <=3 (women)/ >4 and <= 8 (men), >3 and <= 6 (women)/Greater than 8 units (men), greater than 6 units (women) | Alcohol consumption, number of beverages per week (continuous) | Available | Not heterogeneous | Eligible |
| Sodium intake[12] | Not available | Not available | Not available | Not available | Not assessed | Not eligible |
| Menopause[2,4] | Not available | Not available | Menopausal status (categorical): /Regular periods/ Irregular periods/ Periods stopped due to menopause without reproductive surgery/ Surgery | Not available | Not assessed | Not eligible |
| Fluid retention/oedema[2,12] | Not available | Not available | Not available | Not available | Not assessed | Not eligible |
| Adenotonsillar hypertrophy[2] | Not available | Not available | Not available | Not available | Not assessed | Not eligible |
| GERD[8] | Not available | Not available | Not available | Not available | Not assessed | Not eligible |
| Analgesic use[8] | Not available | Not available | Not available | Not available | Not assessed | Not eligible |
| Hypertension[4,7,8,12-15] | 1. Blood pressure:  1.1. Average DBP (continuous)  1.2. Average SBP (continuous)  2. Medications for hypertension: Participant taking any anti-hypertensive medication within two weeks of the SHHS Visit 1 (binary)  3. Diagnosis for hypertension: Hypertension status based on 2nd and 3rd blood pressure readings OR being treated with hypertension meds (binary) | 1. Blood pressure:  1.1. Average DBP (continuous)  1.2. Average SBP (continuous)  2. Medication for hypertension:  2.1. Whether hypertensive: all taking BP drugs (Omron readings) (binary)  2.2. Whether hypertensive:140/90: all prescribed drugs for BP (Omron readings) (binary)  2.3. Diuretics (Blood pressure) (binary)  2.4. Beta blockers (Blood pressure/Fibrinogen) (binary)  2.5. Ace inhibitors (Blood pressure)  2.6. Calcium blockers (Blood pressure) (binary)  2.7. Whether taking drugs prescribed for blood pressure (binary)  2.8 - 2.29. Prescribed Medicines: Reasons for taking medication for high blood pressure (binary)  3. Diagnosis for hypertension: Doctor diagnosed high blood pressure (excluding pregnant) (binary) | 1. Blood pressure:  1.1. Average DBP (continuous)  1.2. Average SBP (continuous)  2. Medications for hypertension (binary)  3. Diagnosis for hypertension by physician (binary) | Available | Not heterogeneous | Eligible |
| Cardiovascular disease[4,12] | 1.HIstory/events of cardiovascular disease:  1.1. Any Cardiovascular Disease (binary) 1.2. Any coronary heart disease (binary) 1.3. Number of myocardial infarctions (continuous) 1.4. Number of congestive heart failures (continuous) 1.5. Number of angina episodes (continuous) | 1. Medications for cardiovascular disease:  1.1. Cardio-vascular medicine taken? (binary) 1.2. Are you currently taking any medicines, tablets or pills for high BP? (binary) | Cardiovascular Disease: Self-reported (binary) | Available | Heterogeneous | Not eligible |
| Diabetes[4,8,16,17] | 1. Medication for diabetes 1.1. Participant taking Oral Hypoglycaemic Agents within two weeks of the SHHS Visit 1. (binary) 1.2. Participants taking insulins within two weeks of the SHHS Visit 1. (binary)  2. Diagnosis for diabetes: History of Diabetes (binary) | 1. Medications for diabetes 1.1. Any prescribed antidiabetic medications taken in last 7 days (binary) 1.2. Antidiabetic prescribed (binary) 1.3. Currently inject insulin for diabetes (binary) 1.4. Currently taking any medicines, tablets or pills for diabetes (binary)  2. Diagnosis/history of diabetes 2.1. Ever had diabetes (binary) 2.2. Total diabetes from blood sample or doctor diagnosis (excluding pregnancy-only diabetes) (binary) | 1: Diabetes Medication/Insulin: Self-reported use (binary)  2: Diabetes: Self-reported diagnosis by a physician (binary) | Available | Not heterogeneous | Eligible |
| Hyperlipaemia[8] | 1. Cholesterol (continuous)  2. Medications for hyperlipaemia: Participant taking any lipid-lowering medication within two weeks of the SHHS Visit 1. (binary) | 1. Valid Total Cholesterol result (continuous)  2. Medications for high cholesterol: 2.1. Lipid lowering (Cholesterol/Fibrinogen) prescribed (binary) 2.2. Any prescribed lipid-lowering medications taken in last 7 days, (binary) | 1: Total cholesterol mg/dL (continuous)  2: Cholesterol Medication: Self-reported use (binary) | Available | Not heterogeneous | Eligible |
| Chronic kidney disease[11,12] | Not available | Not available | Not available | Not available | Not assessed | Not eligible |
| Hypothyroidism[8] | Not available | Not available | Not available | Not available | Not assessed | Not eligible |

Relevant data items were extracted from websites and documents published from the SHHS[21], HSE2019[19], and WSC[20], respectively.
OSA: obstructive sleep apnoea, SHHS: Sleep Heart Health Study, HSE: Health Survey for England, WSC: Wisconsin Sleep Cohort, BMI: body mass index, GERD: Gastroesophageal reflux disease, BP: blood pressure, DBP: diastolic blood pressure, SBP: systolic blood pressure.

### Directed Acyclic Graph (DAG)

We drew a DAG for predictor selection of multiple imputation (Figure S2).[22] The outcome was OSA. Exposure was obesity since its causal pathway to OSA is established.[1-5,7-11] We added risk factors for OSA on the DAG from literature review and clinical experts’ opinions (Table S1), connecting the risk factors with arrows where potential causal relationship is. The DAG suggested adjusting age, ethnicity, occupation, industry, and physical activity (Figure S2). Occupation and industry could be controlled by adjusting ethnicity. As the physical activity data was unavailable, we could not control the physical activity.

Figure S2. A directed acyclic graph (DAG) of causal pathways between obstructive sleep apnoea (OSA) and obesity drawn by DAGitty. Square is outcome, white circle is exposure, and grey circles are confounders. CVD: cardiovascular disease, CKD: chronic kidney disease.

## Supplementary material 3: Data preparation

### Data cleaning and definitions of classifications for variables

We observed distribution, missingness, means, medians, and suspected outliers for continuous data. We used continuous BMI because all datasets contained continuous BMI. To harmonise multiple datasets, we converted other continuous data as categorical as one of the datasets only contains categorical data. For all categorical data, we applied the consistent classifications across all datasets to make the data compatible (Table S2).

### Missing data of HSE2019

In the HSE2019 datasets, data of “Refused”, “Don’t know”, and “Not applicable” for each variable were replaced as missing values. Samples without data of candidate predictor variables were treated as missing data on each variable.

Table S2. Data preparation of variables for multiple imputation model

| **Variables** | **Descriptions** |
| --- | --- |
| AHI | Continuous data of AHI in the SHHS and WSC dataset were converted into binary data so that AHI≥15 can be used as outcome variable for OSA. AHI was classified into either AHI <15 for No OSA or AHI ≥15 for OSA. |
| Age | Age data in all datasets were classified into 0 to 39, 40 to 59, 60 to 64, 65+, then samples aged 0 to 39 and 65+ were excluded across three datasets. As the age data in HSE2019 was categorical, we converted continuous data of age in SHHS and WSC into categorical, which were based on a standard by the Government Statistical Service in the UK (0 to 24, 25 to 64, 65 to 74, and 75+).[23] As the SHHS dataset only contained samples aged 40 years or older, samples below 40 years old were removed from all datasets. To focus on working age, we excluded 65+. Consequently, age categories were 40 to 59, 60 to 64. |
| Sex | Sex data in all datasets were classified into either male or female. |
| Ethnicity | As ethnicity data was highly skewed to white in original three datasets, proportions of other ethnicities were limited, such as black or mixed. Thus, ethnicity data were aggregated into two categories, either white or non-white. |
| BMI | We used continuous BMI data from all datasets. The unit was kg/m^2^. |
| Smoking | Relevant variables about smoking were identified from each dataset and integrated into one variable for each dataset. The SHHS and WSC datasets included variables about smoking experience and current status (i.e. Never, Former, and Current), which were used as smoking classification in this dissertation. The HSE2019 contained some variables about smoking experience and current smoking status including smoking cigarettes, cigars, or pipes (Table S1). Samples who answered “No” to a question about smoking experience were classified as “Never”. Samples who answered “Yes” to at least one question about their current smoking status of cigarettes, cigars, or pipes were classified as “Current”. The rest of samples were classified as “Former”. |
| Alcohol | All datasets contained data related to alcohol intake, though data types were different. The SHHS and WSC contained continuous data, but the HSE2019 contained categorical data. Alcohol data in the SHHS and WSC dataset was converted into binary data. Samples intaking more than zero unit of alcohol per day were classified as “Yes”, and samples who answered zero units of alcohol were classified as “No”. In the SHHS dataset, there were several samples answering more than 50 units of alcoholic drinks per day, which were suspected to be outliers. These samples were classified as “Yes”. In terms of HSE2019, samples who answered “None” to question about number of units were classified as “No”, and samples who drunk more than zero unit were classified as “Yes”. |
| Hypertension | Relevant variables about hypertension were identified from all datasets, including blood pressure, medications for hypertension, and diagnosis or history of hypertension. All datasets contained continuous data about both average diastolic blood pressure (DBP) and average systolic blood pressure (SBP). Samples who showed either more than 90 DBP or more than 140 SBP were classified as “Hypertension”. All datasets contained at least one variable about medications for hypertension (Table S1). Samples who answered “Yes” to at least one question about current medications for hypertension were classified as “Hypertension”. In terms of diagnosis for hypertension, all datasets contained one or two variables about it. Samples who answered “Yes” to at least one question about diagnosis or history of hypertension were classified as “Hypertension”. Samples who did not satisfy any criteria above were classified as “Non-hypertension”. |
| Diabetes | Data related to diabetes, such as diabetic medications and diagnosis for diabetes, were extracted from all datasets. All datasets covered variables about diabetic medications. Samples answering “Yes” among at least one variable about diabetic medication usage were classified as “Diabetes”. Likewise, samples who answered “Yes” to at least one question about diagnosis or history about diabetes were classified as “Diabetes”. Samples who did not satisfy any criteria above were classified as “Non-diabetes”. |
| Hyperlipaemia | Data related to hyperlipaemia were extracted from all datasets. Continuous data of cholesterol was observed in all three datasets, though there was a difference of units (i.e. SHHS: mg/dl, WSC: mg/dl, and HSE2019: mmol/L). Cholesterol data in HSE2019 datasets were converted into mg/dl by multiplying 38.67 to cholesterol data in mmol/L.[24] All datasets contained at least one variable about medications for hyperlipaemia. Samples who answered “Yes” to at least one question about medications for hyperlipaemia were classified as “Hyperlipaemia”. Samples who did not satisfy any criteria above were classified as “Non-hyperlipaemia”. |
| Standard Industry Classification (SIC) | SIC was available in HSE2019 but not in SHHS and WSC. In HSE2019, each participant was asked about current or recent economic activities through individual interview, then the response were classified into SIC groups.[25] The variable name of SIC was ‘SIC2007b’ in the HSE2019 dataset.[26] As there were few participants in industry groups A, B, D, and E, we aggregated them into one (i.e., ABDE). Industry group S was aggregated with Others. |
| Standard Occupation Classification(SOC) | SOC was available in the HSE2019 dataset but not in SHHS and WSC. In the HSE2019, each participant was asked about current or recent occupation through individual interview, then the response was classified into SOC sub-major groups.[25] The variable name of SOC sub-major group was ‘SOC2010B’in the dataset.[26] |
| Survey weights | The SHHS dataset did not provide survey weights data. The SHHS combined participants for six separate studies with complex sampling designs.[27] As it was difficult to estimate weight values of SHHS individuals, we assigned “1” for SHHS participants. |
|  | HSE2019 dataset provided individual weight variable as the survey randomly sampled by weighting sparsely populated area.[25] The variable name was “wt_int”.[26] |
|  | The WSC dataset did not provide survey weights data. The WSC recruited participants high:low risk weighting 1.5:1.[28] The risk was assessed by answers to questions about snoring frequency, loudness of snoring, and witnessed breathing pauses. We identified individual risk by related variables (i.e., snoring frequency: snore_freq, loudness of snoring: snore_vol, and witnessed breathing pauses: choke_freq).[20] If snoring frequency was sometimes or more frequent, or extremely loud, or breathing pauses was sometimes or more, the participant was classified as high risk. We assigned survey weight values of 1.5 on high risk individuals and 1 on low risk individuals. |

## Supplementary material 4: Industries and Occupations

Table S3. Standard Industry Classification 2007 (SIC2007)

| **SIC2007** | | **Short version** |
| --- | --- | --- |
| A | Agriculture, Hunting and fishing | Agriculture and Forestry |
| B | Mining and quarrying | Mining and Quarrying |
| C | Manufacturing | Manufacturing |
| D | Electricity, gas, steam and air conditioning supply | Energy Services |
| E | Water supply; sewerage, waste management and remediation activities | Utilities and Waste |
| F | Construction | Construction |
| G | Wholesale and retail trade; repair of motor vehicles and motor cycles | Retail and Repair |
| H | Transport and storage | Transportation and Storage |
| I | Accommodation and food service activities | Accommodation and Food |
| J | Information and communication | Information and Communication |
| K | Financial and insurance activities | Financial Activities |
| L | Real estate activities | Real Estate |
| M | Professional, scientific and technical activities | Professional Services |
| N | Administrative and support service activities | Administrative Support |
| O | Public administration and defence; compulsory social security | Public Services |
| P | Education | Education |
| Q | Human health and social work activities | Healthcare and Social Work |
| R | Arts, entertainment and recreation | Arts and Entertainment |
| S | Other service activities | Other Services |
| Others | (T) Activities of households etc. | Others |
| SIC2007: Standard Industry Classification 2007 | | |

Table S4. Standard Occupation Classification 2010 (SOC2010)

| **SOC2010** | | **Short version** |
| --- | --- | --- |
| 11 | Corporate managers and directors | Corporate Management |
| 12 | Other managers and proprietors | Other Management |
| 21 | Science, research, engineering and technology professionals | Science and Tech Professionals |
| 22 | Health professionals | Health Professionals |
| 23 | Teaching and educational professionals | Teaching Professionals |
| 24 | Business, media and public service professionals | Business and Media Pros |
| 31 | Science, engineering and technology associate professionals | Tech Associate Pros |
| 32 | Health and social care associate professionals | Health and Social Care Pros |
| 33 | Protective service occupations | Protective Services |
| 34 | Culture, media and sports occupations | Culture and Sports Jobs |
| 35 | Business and public service associate professionals | Business Pros |
| 41 | Administrative occupations | Administrative Jobs |
| 42 | Secretarial and related occupations | Secretarial Jobs |
| 51 | Skilled agricultural and related trades | Agricultural Trades |
| 52 | Skilled metal, electrical and electronic trades | Metal and Electrical Trades |
| 53 | Skilled construction and building trades | Construction Trades |
| 54 | Textiles, printing and other skilled trades | Crafts and Printing |
| 61 | Caring personal service occupations | Caring Services |
| 62 | Leisure, travel and related personal service occupations | Leisure and Travel Services |
| 71 | Sales occupations | Sales Jobs |
| 72 | Customer service occupations | Customer Service |
| 81 | Process, plant and machine operatives | Machine Operatives |
| 82 | Transport and mobile machine drivers and operatives | Transport and Drivers |
| 91 | Elementary trades and related occupations | Elementary Manual Trades |
| 92 | Elementary administration and service occupations | Elementary Administration |
| SOC2010: Sub-Major groups of Standard Occupation Classification 2010 | | |

## Supplementary material 5: Characteristics of study participants

We descriptively compared observed predictors in SHHS and HSE2019 to assess if these datasets have substantial differences. SHHS was 3,122 participants and HSE2019 was 3,437 (Table S5). Age of participants (age 40-64) may be younger in HSE2019 than SHHS. HSE2019 may have more drinker than SHHS. Other categorical variables have no substantial differences among SHHS and HSE2019. Continuous predictor was only BMI, which was comparable between SHHS and HSE2019 from median, mean, and histogram, though the missingness was high in HSE2019 than SHHS (Table S5 and Figure S3).

Table S5. Demographic and anthropometric characteristics of population in the SHHS, the WSC, and the HSE2019 before multiple imputation

| **Characteristic** | **SHHS**,  N = 3,122*^1^* | **HSE2019**,  N = 3,437*^1^* | **WSC**,  N = 910*^1^* |
| --- | --- | --- | --- |
| Age |  |  |  |
| 40-59 | 2,294 (73.5%) | 2,781 (80.9%) | 723 (79.5%) |
| 60-64 | 828 (26.5%) | 656 (19.1%) | 187 (20.5%) |
| NA | 0 | 0 | 0 |
| Sex |  |  |  |
| Female | 1,640 (52.5%) | 1,879 (54.7%) | 432 (47.5%) |
| Male | 1,482 (47.5%) | 1,558 (45.3%) | 478 (52.5%) |
| NA | 0 | 0 | 0 |
| Ethnicity |  |  |  |
| White | 2,537 (81.3%) | 2,927 (85.5%) | 862 (94.7%) |
| Non White | 585 (18.7%) | 497 (14.5%) | 48 (5.3%) |
| NA | 0 | 13 | 0 |
| BMI |  |  |  |
| Median, Mean(SD) | 27.8, 28.5(5.4) | 27.6, 28.5(5.7) | 30.0, 31.8(7.4) |
| NA | 37 | 597 | 0 |
| Smoking |  |  |  |
| Never | 1,446 (46.8%) | 1,624 (47.4%) | 462 (50.8%) |
| Former | 1,260 (40.7%) | 1,241 (36.2%) | 341 (37.5%) |
| Current | 387 (12.5%) | 559 (16.3%) | 107 (11.8%) |
| NA | 29 | 13 | 0 |
| Alcohol |  |  |  |
| Non Drinker | 1,380 (49.6%) | 1,363 (39.8%) | 320 (35.2%) |
| Drinker | 1,404 (50.4%) | 2,061 (60.2%) | 590 (64.8%) |
| NA | 338 | 13 | 0 |
| Hypertension |  |  |  |
| Non Hypertension | 1,948 (62.4%) | 2,363 (68.8%) | 516 (56.7%) |
| Hypertension | 1,174 (37.6%) | 1,074 (31.2%) | 394 (43.3%) |
| NA | 0 | 0 | 0 |
| Diabetes |  |  |  |
| Non Diabetes | 2,972 (95.3%) | 3,106 (90.4%) | 848 (93.2%) |
| Diabetes | 147 (4.7%) | 328 (9.6%) | 62 (6.8%) |
| NA | 3 | 3 | 0 |
| AHI |  |  |  |
| Median, Mean(SD) | 4.0, 8.9(13.3) | NA, NA(NA) | 5.1, 10.8(14.9) |
| NA | 0 | 3,437 | 0 |
| OSA |  |  |  |
| Non OSA_or Mild_OSA | 2,572 (82.4%) | 0 (NA%) | 699 (76.8%) |
| Moderate to Severe_OSA | 550 (17.6%) | 0 (NA%) | 211 (23.2%) |
| NA | 0 | 3,437 | 0 |
| SIC2007 |  |  |  |
| (ABDE) Agriculture, Mining, Energy, Utilities & Waste | 0 (NA%) | 79 (2.4%) | 0 (NA%) |
| (C) Manufacturing | 0 (NA%) | 343 (10.3%) | 0 (NA%) |
| (F) Construction | 0 (NA%) | 256 (7.7%) | 0 (NA%) |
| (G) Retail and Repair | 0 (NA%) | 399 (12.0%) | 0 (NA%) |
| (H) Transportation and Storage | 0 (NA%) | 246 (7.4%) | 0 (NA%) |
| (I) Accommodation and Food | 0 (NA%) | 121 (3.6%) | 0 (NA%) |
| (J) Information and Communication | 0 (NA%) | 108 (3.3%) | 0 (NA%) |
| (K) Financial Activities | 0 (NA%) | 92 (2.8%) | 0 (NA%) |
| (L) Real Estate | 0 (NA%) | 23 (0.7%) | 0 (NA%) |
| (M) Professional Services | 0 (NA%) | 210 (6.3%) | 0 (NA%) |
| (N) Administrative Support | 0 (NA%) | 193 (5.8%) | 0 (NA%) |
| (O) Public Services | 0 (NA%) | 201 (6.0%) | 0 (NA%) |
| (P) Education | 0 (NA%) | 388 (11.7%) | 0 (NA%) |
| (Q) Healthcare and Social Work | 0 (NA%) | 507 (15.3%) | 0 (NA%) |
| (R) Arts and Entertainment | 0 (NA%) | 65 (2.0%) | 0 (NA%) |
| (Others) Others | 0 (NA%) | 92 (2.8%) | 0 (NA%) |
| NA | 3,122 | 114 | 910 |
| SOC2010_Sub_category |  |  |  |
| (11) Corporate Management | 0 (NA%) | 235 (7.1%) | 0 (NA%) |
| (12) Other Management | 0 (NA%) | 117 (3.5%) | 0 (NA%) |
| (21) Science and Tech Professionals | 0 (NA%) | 142 (4.3%) | 0 (NA%) |
| (22) Health Professionals | 0 (NA%) | 144 (4.3%) | 0 (NA%) |
| (23) Teaching Professionals | 0 (NA%) | 162 (4.9%) | 0 (NA%) |
| (24) Business and Media Pros | 0 (NA%) | 160 (4.8%) | 0 (NA%) |
| (31) Tech Associate Pros | 0 (NA%) | 41 (1.2%) | 0 (NA%) |
| (32) Health and Social Care Pros | 0 (NA%) | 46 (1.4%) | 0 (NA%) |
| (33) Protective Services | 0 (NA%) | 38 (1.1%) | 0 (NA%) |
| (34) Culture and Sports Jobs | 0 (NA%) | 63 (1.9%) | 0 (NA%) |
| (35) Business Pros | 0 (NA%) | 221 (6.7%) | 0 (NA%) |
| (41) Administrative Jobs | 0 (NA%) | 309 (9.3%) | 0 (NA%) |
| (42) Secretarial Jobs | 0 (NA%) | 99 (3.0%) | 0 (NA%) |
| (51) Agricultural Trades | 0 (NA%) | 43 (1.3%) | 0 (NA%) |
| (52) Metal and Electrical Trades | 0 (NA%) | 108 (3.3%) | 0 (NA%) |
| (53) Construction Trades | 0 (NA%) | 119 (3.6%) | 0 (NA%) |
| (54) Crafts and Printing | 0 (NA%) | 71 (2.1%) | 0 (NA%) |
| (61) Caring Services | 0 (NA%) | 280 (8.4%) | 0 (NA%) |
| (62) Leisure and Travel Services | 0 (NA%) | 73 (2.2%) | 0 (NA%) |
| (71) Sales Jobs | 0 (NA%) | 174 (5.2%) | 0 (NA%) |
| (72) Customer Service | 0 (NA%) | 37 (1.1%) | 0 (NA%) |
| (81) Machine Operatives | 0 (NA%) | 134 (4.0%) | 0 (NA%) |
| (82) Transport and Drivers | 0 (NA%) | 141 (4.2%) | 0 (NA%) |
| (91) Elementary Manual Trades | 0 (NA%) | 49 (1.5%) | 0 (NA%) |
| (92) Elementary Administration | 0 (NA%) | 317 (9.5%) | 0 (NA%) |
| NA | 3,122 | 114 | 910 |
| *^1^*n (%), NA: Missing data, SHHS: Sleep Heart Health Study, HSE: Health Survey for England, WSC: Wisconsin Sleep Cohort, AHI: Apnoea Hypopnoea Index, OSA: Obstructive Sleep Apnoea, SIC: Standard Industry Classification, SOC: Standard Occupation Classification | | | |

Figure S3. A histogram of BMI in SHHS and HSE2019 aged 40-64. SHHS: Sleep Heart Health Study Visit 1, HSE2019: Health Survey for England 2019.

### MAR assumption

After omitting participants aged<40 and >64, BMI contained large missingness in HSE2019 (597, Table S5). The HSE2019 dataset provided reasons for missingness in BMI (variable name: ‘BMIOK’).[19] Among participants aged 40-64, major reasons for missingness in BMI included *height/weight not attempted* (n=240, 7.5%) and *refusal* (n=303, 8.8%). From the document protocol, the weight scale limit was 200kg, and if the investigator thought that the participant’s weight exceeds 200kg, they record “not attempted” for the participant.[25] However, the dataset did not show the reasons of “not attempted” for each participant, but it is unreasonable to assume that the proportion of weight>200kg is 7.5%. From the information described above, it would be reasonable to conclude that the missingness in weight might not depend on their values. There was not enough information to assume MNAR for BMI, so we concluded that the missingness in BMI was MAR in the combined dataset.

## Supplementary material 6: Multiple imputation model

Table S6. Pooled estimates of multiple imputation models for OSA in combined dataset with SHHS and HSE2019 (age 40-64)

| **Characteristic** | **OR**^1^ | **95% CI**^1^ | **p-value** |
| --- | --- | --- | --- |
| Age |  |  |  |
| Age 40-59 | — | — |  |
| Age 60-64 | 1.21 | 1.05, 1.40 | 0.008 |
| Sex |  |  |  |
| Female | — | — |  |
| Male | 1.60 | 1.40, 1.81 | <0.001 |
| Ethnicity |  |  |  |
| White | — | — |  |
| Non White | 1.01 | 0.80, 1.27 | 0.940 |
| BMI | 1.06 | 1.05, 1.08 | <0.001 |
| Smoking |  |  |  |
| Never | — | — |  |
| Former | 0.91 | 0.75, 1.11 | 0.345 |
| Current | 0.92 | 0.80, 1.07 | 0.299 |
| Alcohol |  |  |  |
| Non Drinker | — | — |  |
| Drinker | 0.93 | 0.78, 1.10 | 0.382 |
| Hypertension |  |  |  |
| Non Hypertension | — | — |  |
| Hypertension | 1.21 | 1.07, 1.38 | 0.004 |
| Diabetes |  |  |  |
| Non Diabetes | — | — |  |
| Diabetes | 0.85 | 0.63, 1.16 | 0.316 |
| SIC2007 |  |  |  |
| (ABDE) Agriculture, Mining, Energy, Utilities & Waste | — | — |  |
| (C) Manufacturing | 1.05 | 0.49, 2.25 | 0.896 |
| (F) Construction | 1.03 | 0.46, 2.27 | 0.946 |
| (G) Retail and Repair | 1.08 | 0.53, 2.21 | 0.831 |
| (H) Transportation and Storage | 0.96 | 0.41, 2.22 | 0.918 |
| (I) Accommodation and Food | 1.17 | 0.55, 2.49 | 0.676 |
| (J) Information and Communication | 1.08 | 0.52, 2.26 | 0.831 |
| (K) Financial Activities | 1.11 | 0.45, 2.77 | 0.811 |
| (L) Real Estate | 0.97 | 0.21, 4.39 | 0.967 |
| (M) Professional Services | 1.14 | 0.56, 2.33 | 0.707 |
| (N) Administrative Support | 1.01 | 0.48, 2.14 | 0.972 |
| (O) Public Services | 1.14 | 0.57, 2.28 | 0.710 |
| (P) Education | 0.92 | 0.43, 1.97 | 0.817 |
| (Q) Healthcare and Social Work | 1.07 | 0.55, 2.10 | 0.831 |
| (R) Arts and Entertainment | 0.93 | 0.39, 2.19 | 0.858 |
| (Others) Others | 1.00 | 0.40, 2.51 | >0.999 |
| SOC2010_Sub_category |  |  |  |
| (11) Corporate Management | — | — |  |
| (12) Other Management | 1.11 | 0.42, 2.91 | 0.825 |
| (21) Science and Tech Professionals | 0.91 | 0.47, 1.75 | 0.777 |
| (22) Health Professionals | 1.15 | 0.62, 2.11 | 0.661 |
| (23) Teaching Professionals | 1.17 | 0.66, 2.09 | 0.581 |
| (24) Business and Media Pros | 0.85 | 0.47, 1.53 | 0.572 |
| (31) Tech Associate Pros | 0.76 | 0.20, 2.86 | 0.682 |
| (32) Health and Social Care Pros | 1.47 | 0.50, 4.31 | 0.475 |
| (33) Protective Services | 1.26 | 0.45, 3.50 | 0.653 |
| (34) Culture and Sports Jobs | 1.20 | 0.57, 2.49 | 0.629 |
| (35) Business Pros | 1.21 | 0.70, 2.09 | 0.496 |
| (41) Administrative Jobs | 1.14 | 0.63, 2.06 | 0.650 |
| (42) Secretarial Jobs | 1.58 | 0.74, 3.38 | 0.234 |
| (51) Agricultural Trades | 1.02 | 0.44, 2.34 | 0.970 |
| (52) Metal and Electrical Trades | 0.75 | 0.26, 2.21 | 0.588 |
| (53) Construction Trades | 1.00 | 0.46, 2.21 | 0.995 |
| (54) Crafts and Printing | 0.99 | 0.30, 3.25 | 0.988 |
| (61) Caring Services | 1.17 | 0.63, 2.16 | 0.609 |
| (62) Leisure and Travel Services | 1.07 | 0.56, 2.07 | 0.827 |
| (71) Sales Jobs | 1.01 | 0.58, 1.76 | 0.982 |
| (72) Customer Service | 0.93 | 0.24, 3.51 | 0.908 |
| (81) Machine Operatives | 1.05 | 0.62, 1.78 | 0.849 |
| (82) Transport and Drivers | 1.06 | 0.58, 1.92 | 0.850 |
| (91) Elementary Manual Trades | 1.03 | 0.56, 1.89 | 0.936 |
| (92) Elementary Administration | 1.00 | 0.56, 1.80 | 0.995 |
| ^1^OR = Odds Ratio, CI = Confidence Interval | | | |

### Multiple imputation model for process validation

Table S7. Pooled estimates of multiple imputation models for OSA in combined dataset with SHHS and WSC (age 40-64)

| **Characteristic** | **OR**^1^ | **95% CI**^1^ | **p-value** |
| --- | --- | --- | --- |
| Age |  |  |  |
| Age 40-59 | — | — |  |
| Age 60-64 | 1.42 | 1.22, 1.65 | <0.001 |
| Sex |  |  |  |
| Female | — | — |  |
| Male | 2.02 | 1.76, 2.31 | <0.001 |
| Ethnicity |  |  |  |
| White | — | — |  |
| Non White | 1.08 | 0.81, 1.43 | 0.595 |
| BMI | 1.08 | 1.06, 1.10 | <0.001 |
| Smoking |  |  |  |
| Never | — | — |  |
| Former | 0.93 | 0.75, 1.16 | 0.523 |
| Current | 0.92 | 0.78, 1.08 | 0.319 |
| Alcohol |  |  |  |
| Non Drinker | — | — |  |
| Drinker | 0.90 | 0.74, 1.10 | 0.320 |
| Hypertension |  |  |  |
| Non Hypertension | — | — |  |
| Hypertension | 1.27 | 1.10, 1.45 | <0.001 |
| Diabetes |  |  |  |
| Non Diabetes | — | — |  |
| Diabetes | 0.87 | 0.55, 1.37 | 0.545 |
| ^1^OR = Odds Ratio, CI = Confidence Interval,  SHHS = Sleep Heart Health Study, WSC = Wisconsin Sleep Cohort | | | |

## Supplementary material 7: Pooled summary characteristics of study participants

Table S8. Pooled summary characteristics from ten-imputed datasets in HSE2019 (age 40-64)

| **Variables** | | **All participants** | | | **Participants with OSA** | | | **Participants without OSA** | | | **p-value^1^** |
| --- | --- | --- | --- | --- | --- | --- | --- | --- | --- | --- | --- |
|  |  | **Estimates** | **Lower CI** | **Upper CI** | **Estimates** | **Lower CI** | **Upper CI** | **Estimates** | **Lower CI** | **Upper CI** |  |
| Age |  |  |  |  |  |  |  |  |  |  | 0.515 |
|  | Age40-59 | 80.9% | 80.9% | 80.9% | 81.5% | 77.1% | 85.3% | 80.8% | 79.2% | 82.3% |  |
|  | Age60-64 | 19.1% | 19.1% | 19.1% | 18.5% | 14.7% | 22.9% | 19.2% | 17.7% | 20.8% |  |
| Sex |  |  |  |  |  |  |  |  |  |  | 0.463 |
|  | Female | 54.7% | 54.7% | 54.7% | 53.7% | 48.4% | 59.0% | 54.9% | 52.9% | 56.9% |  |
|  | Male | 45.3% | 45.3% | 45.3% | 46.3% | 41.0% | 51.6% | 45.1% | 43.1% | 47.1% |  |
| Ethnicity |  |  |  |  |  |  |  |  |  |  | 0.711 |
|  | White | 85.5% | 84.3% | 86.6% | 84.8% | 81.0% | 87.9% | 85.6% | 84.2% | 87.0% |  |
|  | Non white | 14.5% | 13.4% | 15.7% | 15.3% | 12.1% | 19.0% | 14.4% | 13.0% | 15.8% |  |
| BMI |  |  |  |  |  |  |  |  |  |  | 0.749 |
|  | Mean (95%CI) | 28.52 | 28.31 | 28.72 | 28.61 | 27.94 | 29.29 | 28.5 | 28.27 | 28.72 |  |
| Smoking |  |  |  |  |  |  |  |  |  |  | 0.871 |
|  | Never smoking | 47.4% | 45.7% | 49.1% | 47.6% | 42.8% | 52.6% | 47.4% | 45.4% | 49.3% |  |
|  | Former smoker | 36.3% | 34.7% | 37.9% | 36.4% | 31.6% | 41.5% | 36.2% | 34.3% | 38.2% |  |
|  | Current smoker | 16.3% | 15.1% | 17.6% | 16.0% | 12.5% | 20.2% | 16.4% | 15.0% | 18.0% |  |
| Alcohol |  |  |  |  |  |  |  |  |  |  | 0.386 |
|  | Non drinker | 39.9% | 38.2% | 41.5% | 42.1% | 37.2% | 47.2% | 39.4% | 37.5% | 41.3% |  |
|  | Drinker | 60.1% | 58.5% | 61.8% | 57.9% | 52.8% | 62.8% | 60.6% | 58.7% | 62.5% |  |
| Hypertension | |  |  |  |  |  |  |  |  |  | 0.552 |
|  | Non hypertension | 68.8% | 68.8% | 68.8% | 68.2% | 62.6% | 73.2% | 68.9% | 66.9% | 70.7% |  |
|  | Hypertension | 31.2% | 31.2% | 31.2% | 31.8% | 26.8% | 37.4% | 31.1% | 29.3% | 33.1% |  |
| Diabetes |  |  |  |  |  |  |  |  |  |  | 0.567 |
|  | Non diabetes | 90.4% | 89.4% | 91.4% | 90.2% | 86.3% | 93.1% | 90.5% | 89.2% | 91.6% |  |
|  | Diabetes | 9.6% | 8.6% | 10.6% | 9.8% | 6.9% | 13.7% | 9.5% | 8.4% | 10.8% |  |
| OSA |  |  |  |  |  |  |  |  |  |  | <0.001 |
|  | Non OSA | 82.2% | 80.1% | 84.1% | 0.0% | 0.0% | 0.6% | 100.0% | 100.0% | 100.0% |  |
|  | OSA | 17.8% | 15.9% | 19.9% | 100.0% | 100.0% | 100.0% | 0.0% | 0.0% | 0.1% |  |
| SIC2007 |  |  |  |  |  |  |  |  |  |  | 0.980 |
|  | (ABDE) Agriculture, Mining, Energy, Utilities & Waste | 2.4% | 1.9% | 2.9% | 2.5% | 1.4% | 4.6% | 2.3% | 1.8% | 3.0% |  |
|  | (C) Manufacturing | 10.4% | 9.4% | 11.5% | 10.2% | 7.3% | 14.0% | 10.4% | 9.2% | 11.8% |  |
|  | (F) Construction | 7.8% | 6.9% | 8.7% | 8.4% | 5.9% | 11.9% | 7.6% | 6.6% | 8.8% |  |
|  | (G) Retail and Repair | 11.9% | 10.8% | 13.0% | 11.6% | 9.0% | 14.7% | 11.9% | 10.7% | 13.3% |  |
|  | (H) Transportation and Storage | 7.4% | 6.6% | 8.3% | 7.4% | 5.3% | 10.3% | 7.4% | 6.4% | 8.5% |  |
|  | (I) Accommodation and Food | 3.6% | 3.1% | 4.3% | 4.2% | 2.7% | 6.4% | 3.5% | 2.9% | 4.3% |  |
|  | (J) Information and Communication | 3.3% | 2.7% | 3.9% | 3.2% | 1.9% | 5.4% | 3.3% | 2.6% | 4.1% |  |
|  | (K) Financial Activities | 2.8% | 2.3% | 3.4% | 3.0% | 1.6% | 5.4% | 2.7% | 2.1% | 3.5% |  |
|  | (L) Real Estate | 0.7% | 0.5% | 1.0% | 0.6% | 0.2% | 1.7% | 0.7% | 0.5% | 1.1% |  |
|  | (M) Professional Services | 6.4% | 5.6% | 7.3% | 6.2% | 4.3% | 8.7% | 6.4% | 5.5% | 7.4% |  |
|  | (N) Administrative Support | 5.7% | 5.0% | 6.6% | 6.1% | 4.0% | 9.3% | 5.7% | 4.8% | 6.7% |  |
|  | (O) Public Services | 6.0% | 5.2% | 6.9% | 6.6% | 4.5% | 9.6% | 5.9% | 5.0% | 6.9% |  |
|  | (P) Education | 11.6% | 10.6% | 12.8% | 10.1% | 7.7% | 13.1% | 12.0% | 10.8% | 13.3% |  |
|  | (Q) Healthcare and Social Work | 15.4% | 14.2% | 16.6% | 15.6% | 12.5% | 19.4% | 15.3% | 14.0% | 16.7% |  |
|  | (R) Arts and Entertainment | 2.0% | 1.5% | 2.5% | 1.8% | 0.8% | 3.7% | 2.0% | 1.5% | 2.6% |  |
|  | (Others) Others | 2.8% | 2.3% | 3.4% | 2.5% | 1.3% | 4.6% | 2.8% | 2.2% | 3.6% |  |
| SOC2010 |  |  |  |  |  |  |  |  |  |  | 0.985 |
|  | (11) Corporate Management | 6.8% | 6.1% | 7.7% | 7.0% | 4.6% | 10.4% | 6.8% | 5.9% | 7.9% |  |
|  | (12) Other Management | 3.4% | 2.9% | 4.1% | 3.4% | 1.9% | 6.0% | 3.4% | 2.7% | 4.2% |  |
|  | (21) Science and Tech Professionals | 4.3% | 3.6% | 5.0% | 4.4% | 2.7% | 7.1% | 4.2% | 3.5% | 5.1% |  |
|  | (22) Health Professionals | 4.3% | 3.6% | 5.0% | 3.8% | 2.2% | 6.6% | 4.4% | 3.6% | 5.3% |  |
|  | (23) Teaching Professionals | 5.1% | 4.4% | 5.8% | 4.5% | 2.7% | 7.4% | 5.2% | 4.4% | 6.1% |  |
|  | (24) Business and Media Pros | 5.0% | 4.3% | 5.8% | 4.5% | 2.7% | 7.4% | 5.1% | 4.3% | 6.1% |  |
|  | (31) Tech Associate Pros | 1.2% | 0.9% | 1.6% | 1.1% | 0.4% | 3.0% | 1.2% | 0.9% | 1.8% |  |
|  | (32) Health and Social Care Pros | 1.4% | 1.0% | 1.8% | 1.7% | 0.8% | 3.6% | 1.3% | 0.9% | 1.8% |  |
|  | (33) Protective Services | 1.1% | 0.8% | 1.5% | 1.4% | 0.7% | 2.8% | 1.0% | 0.7% | 1.5% |  |
|  | (34) Culture and Sports Jobs | 1.9% | 1.5% | 2.4% | 1.7% | 0.8% | 3.3% | 1.9% | 1.4% | 2.5% |  |
|  | (35) Business Pros | 6.6% | 5.8% | 7.4% | 6.8% | 4.8% | 9.7% | 6.5% | 5.6% | 7.5% |  |
|  | (41) Administrative Jobs | 9.2% | 8.3% | 10.2% | 8.8% | 6.4% | 12.0% | 9.3% | 8.2% | 10.5% |  |
|  | (42) Secretarial Jobs | 2.9% | 2.4% | 3.5% | 3.3% | 2.0% | 5.2% | 2.8% | 2.3% | 3.6% |  |
|  | (51) Agricultural Trades | 1.3% | 1.0% | 1.7% | 1.3% | 0.6% | 2.9% | 1.3% | 0.9% | 1.8% |  |
|  | (52) Metal and Electrical Trades | 3.2% | 2.6% | 3.8% | 3.1% | 1.6% | 5.7% | 3.2% | 2.5% | 4.0% |  |
|  | (53) Construction Trades | 3.5% | 2.9% | 4.1% | 4.2% | 2.5% | 6.8% | 3.3% | 2.7% | 4.1% |  |
|  | (54) Crafts and Printing | 2.1% | 2.1% | 2.1% | 2.3% | 1.2% | 4.3% | 2.0% | 1.5% | 2.7% |  |
|  | (61) Caring Services | 8.5% | 7.6% | 9.5% | 8.7% | 6.6% | 11.4% | 8.4% | 7.4% | 9.5% |  |
|  | (62) Leisure and Travel Services | 2.3% | 1.8% | 2.8% | 2.2% | 1.0% | 4.4% | 2.3% | 1.8% | 3.0% |  |
|  | (71) Sales Jobs | 5.5% | 4.8% | 6.3% | 5.7% | 3.9% | 8.3% | 5.4% | 4.6% | 6.4% |  |
|  | (72) Customer Service | 1.1% | 0.8% | 1.5% | 1.1% | 0.4% | 2.7% | 1.1% | 0.7% | 1.6% |  |
|  | (81) Machine Operatives | 4.4% | 3.7% | 5.1% | 5.1% | 3.3% | 7.6% | 4.2% | 3.5% | 5.1% |  |
|  | (82) Transport and Drivers | 4.3% | 3.7% | 5.1% | 4.2% | 2.5% | 6.8% | 4.4% | 3.6% | 5.3% |  |
|  | (91) Elementary Manual Trades | 1.6% | 1.2% | 2.1% | 1.3% | 0.5% | 3.3% | 1.6% | 1.2% | 2.2% |  |
|  | (92) Elementary Administration | 9.4% | 8.4% | 10.4% | 8.6% | 6.2% | 11.9% | 9.5% | 8.4% | 10.8% |  |

^1^ Chi-squared test by pool_D2()[29] for other variables, Unpaired student t-test by pool_t_test()[30] for BMI, CI: 95% confidence interval,
OSA: moderate to severe OSA (AHI≥15)

## Supplementary material 8: Estimated prevalence of OSA by industries in England

Table S9. Estimated prevalence of OSA by industries in England in 2019 (age 40-64)

| **SIC2007** | **Estimates** | **Lower CI** | **Upper CI** |
| --- | --- | --- | --- |
| (ABDE) Agriculture, Mining, Energy, Utilities & Waste | 18.9% | 10.3% | 32.0% |
| (C) Manufacturing | 17.4% | 12.1% | 24.4% |
| (F) Construction | 19.4% | 13.3% | 27.4% |
| (G) Retail and Repair | 17.3% | 13.2% | 22.4% |
| (H) Transportation and Storage | 17.8% | 12.5% | 24.7% |
| (I) Accommodation and Food | 20.5% | 13.1% | 30.8% |
| (J) Information and Communication | 17.6% | 10.2% | 28.7% |
| (K) Financial Activities | 19.3% | 10.3% | 33.1% |
| (L) Real Estate | 14.7% | 4.6% | 38.3% |
| (M) Professional Services | 17.2% | 12.0% | 24.0% |
| (N) Administrative Support | 19.0% | 12.7% | 27.4% |
| (O) Public Services | 19.7% | 13.0% | 28.7% |
| (P) Education | 15.5% | 11.5% | 20.5% |
| (Q) Healthcare and Social Work | 18.1% | 14.5% | 22.5% |
| (R) Arts and Entertainment | 16.1% | 7.7% | 30.7% |
| (Others) Others | 15.9% | 8.7% | 27.3% |
| SIC2007: Standard Industry Classification 2007, CI: 95% confidence interval | | | |

Table S10. Pooled differences of proportions of OSA between industries in England in 2019 (age 40-64)

|  | **Estimates, Lower CI, Upper CI** | | | | | | | | | | | | | | | | | | | | | | | |
| --- | --- | --- | --- | --- | --- | --- | --- | --- | --- | --- | --- | --- | --- | --- | --- | --- | --- | --- | --- | --- | --- | --- | --- | --- |
| **SIC2007** | **ABDE** | | | **C** | | | **F** | | | **G** | | | **H** | | | **I** | | | **J** | | | **K** | | |
| **ABDE** |  |  |  | 1.4% | -9.6% | 15.5% | -0.5% | -12.2% | 13.9% | 1.6% | -8.3% | 15.2% | 1.1% | -9.9% | 15.2% | -1.7% | -14.9% | 13.4% | 1.3% | -12.7% | 16.3% | -0.4% | -16.6% | 15.4% |
| **C** | -1.4% | -15.5% | 9.6% |  |  |  | -2.0% | -11.6% | 7.3% | 0.1% | -7.2% | 8.2% | -0.4% | -9.1% | 8.4% | -3.1% | -14.6% | 7.1% | -0.2% | -12.4% | 10.0% | -1.8% | -16.6% | 9.5% |
| **F** | 0.5% | -13.9% | 12.2% | 2.0% | -7.3% | 11.6% |  |  |  | 2.1% | -5.8% | 11.1% | 1.6% | -7.6% | 11.2% | -1.2% | -13.0% | 9.8% | 1.8% | -10.8% | 12.7% | 0.1% | -14.9% | 12.1% |
| **G** | -1.6% | -15.2% | 8.3% | -0.1% | -8.2% | 7.2% | -2.1% | -11.1% | 5.8% |  |  |  | -0.5% | -8.5% | 6.8% | -3.2% | -14.2% | 5.8% | -0.3% | -12.1% | 8.6% | -1.9% | -16.3% | 8.3% |
| **H** | -1.1% | -15.2% | 9.9% | 0.4% | -8.4% | 9.1% | -1.6% | -11.2% | 7.6% | 0.5% | -6.8% | 8.5% |  |  |  | -2.7% | -14.2% | 7.4% | 0.2% | -12.1% | 10.3% | -1.5% | -16.2% | 9.8% |
| **I** | 1.7% | -13.4% | 14.9% | 3.1% | -7.1% | 14.6% | 1.2% | -9.8% | 13.0% | 3.2% | -5.8% | 14.2% | 2.7% | -7.4% | 14.2% |  |  |  | 2.9% | -10.4% | 15.5% | 1.3% | -14.4% | 14.8% |
| **J** | -1.3% | -16.3% | 12.7% | 0.2% | -10.0% | 12.4% | -1.8% | -12.7% | 10.8% | 0.3% | -8.6% | 12.1% | -0.2% | -10.3% | 12.1% | -2.9% | -15.5% | 10.4% |  |  |  | -1.6% | -17.3% | 12.5% |
| **K** | 0.4% | -15.4% | 16.6% | 1.8% | -9.5% | 16.6% | -0.1% | -12.1% | 14.9% | 1.9% | -8.3% | 16.3% | 1.5% | -9.8% | 16.2% | -1.3% | -14.8% | 14.4% | 1.6% | -12.5% | 17.3% |  |  |  |
| **L** | -4.2% | -20.7% | 20.3% | -2.8% | -15.0% | 20.9% | -4.7% | -17.5% | 19.1% | -2.6% | -13.8% | 20.7% | -3.1% | -15.3% | 20.5% | -5.9% | -20.1% | 18.3% | -2.9% | -17.8% | 21.2% | -4.6% | -21.6% | 20.1% |
| **M** | -1.7% | -15.7% | 9.2% | -0.3% | -9.0% | 8.4% | -2.2% | -11.8% | 6.9% | -0.1% | -7.4% | 7.8% | -0.6% | -9.3% | 8.0% | -3.4% | -14.8% | 6.7% | -0.4% | -12.6% | 9.6% | -2.1% | -16.8% | 9.1% |
| **N** | 0.1% | -14.4% | 12.1% | 1.5% | -7.9% | 11.5% | -0.4% | -10.6% | 10.0% | 1.7% | -6.4% | 11.0% | 1.2% | -8.2% | 11.1% | -1.6% | -13.5% | 9.7% | 1.4% | -11.4% | 12.6% | -0.3% | -15.4% | 12.0% |
| **O** | 0.8% | -13.9% | 13.2% | 2.2% | -7.4% | 12.7% | 0.3% | -10.1% | 11.1% | 2.4% | -6.0% | 12.2% | 1.9% | -7.7% | 12.3% | -0.9% | -13.0% | 10.8% | 2.1% | -10.8% | 13.7% | 0.4% | -14.9% | 13.1% |
| **P** | -3.4% | -17.0% | 6.5% | -2.0% | -10.0% | 5.3% | -3.9% | -12.9% | 4.0% | -1.8% | -8.2% | 4.6% | -2.3% | -10.3% | 5.0% | -5.1% | -16.0% | 3.9% | -2.2% | -13.9% | 6.8% | -3.8% | -18.1% | 6.4% |
| **Q** | -0.8% | -14.3% | 8.8% | 0.7% | -7.2% | 7.6% | -1.3% | -10.1% | 6.2% | 0.8% | -5.4% | 6.8% | 0.3% | -7.5% | 7.2% | -2.4% | -13.2% | 6.2% | 0.5% | -11.1% | 9.1% | -1.1% | -15.4% | 8.8% |
| **R** | -2.8% | -18.3% | 14.0% | -1.4% | -12.3% | 14.1% | -3.3% | -14.9% | 12.4% | -1.2% | -11.0% | 13.8% | -1.7% | -12.6% | 13.7% | -4.5% | -17.6% | 11.8% | -1.5% | -15.4% | 14.7% | -3.2% | -19.3% | 13.8% |
| **Others** | -3.0% | -17.9% | 11.2% | -1.6% | -11.6% | 11.0% | -3.5% | -14.3% | 9.4% | -1.4% | -10.2% | 10.6% | -1.9% | -11.9% | 10.6% | -4.7% | -17.1% | 8.9% | -1.8% | -14.9% | 11.8% | -3.4% | -18.9% | 11.1% |
|  |  |  |  |  |  |  |  |  |  |  |  |  |  |  |  |  |  |  |  |  |  |  |  |  |
|  | **Estimates, Lower CI, Upper CI** | | | | | | | | | | | | | | | | | | | | | | | |
| **SIC2007** | **L** | | | **M** | | | **N** | | | **O** | | | **P** | | | **Q** | | | **R** | | | **Others** | | |
| **ABDE** | 4.2% | -20.3% | 20.7% | 1.7% | -9.2% | 15.7% | -0.1% | -12.1% | 14.4% | -0.8% | -13.2% | 13.9% | 3.4% | -6.5% | 17.0% | 0.8% | -8.8% | 14.3% | 2.8% | -14.0% | 18.3% | 3.0% | -11.2% | 17.9% |
| **C** | 2.8% | -20.9% | 15.0% | 0.3% | -8.4% | 9.0% | -1.5% | -11.5% | 7.9% | -2.2% | -12.7% | 7.4% | 2.0% | -5.3% | 10.0% | -0.7% | -7.6% | 7.2% | 1.4% | -14.1% | 12.3% | 1.6% | -11.0% | 11.6% |
| **F** | 4.7% | -19.1% | 17.5% | 2.2% | -6.9% | 11.8% | 0.4% | -10.0% | 10.6% | -0.3% | -11.1% | 10.1% | 3.9% | -4.0% | 12.9% | 1.3% | -6.2% | 10.1% | 3.3% | -12.4% | 14.9% | 3.5% | -9.4% | 14.3% |
| **G** | 2.6% | -20.7% | 13.8% | 0.1% | -7.8% | 7.4% | -1.7% | -11.0% | 6.4% | -2.4% | -12.2% | 6.0% | 1.8% | -4.6% | 8.2% | -0.8% | -6.8% | 5.4% | 1.2% | -13.8% | 11.0% | 1.4% | -10.6% | 10.2% |
| **H** | 3.1% | -20.5% | 15.3% | 0.6% | -8.0% | 9.3% | -1.2% | -11.1% | 8.2% | -1.9% | -12.3% | 7.7% | 2.3% | -5.0% | 10.3% | -0.3% | -7.2% | 7.5% | 1.7% | -13.7% | 12.6% | 1.9% | -10.6% | 11.9% |
| **I** | 5.9% | -18.3% | 20.1% | 3.4% | -6.7% | 14.8% | 1.6% | -9.7% | 13.5% | 0.9% | -10.8% | 13.0% | 5.1% | -3.9% | 16.0% | 2.4% | -6.2% | 13.2% | 4.5% | -11.8% | 17.6% | 4.7% | -8.9% | 17.1% |
| **J** | 2.9% | -21.2% | 17.8% | 0.4% | -9.6% | 12.6% | -1.4% | -12.6% | 11.4% | -2.1% | -13.7% | 10.8% | 2.2% | -6.8% | 13.9% | -0.5% | -9.1% | 11.1% | 1.5% | -14.7% | 15.4% | 1.8% | -11.8% | 14.9% |
| **K** | 4.6% | -20.1% | 21.6% | 2.1% | -9.1% | 16.8% | 0.3% | -12.0% | 15.4% | -0.4% | -13.1% | 14.9% | 3.8% | -6.4% | 18.1% | 1.1% | -8.8% | 15.4% | 3.2% | -13.8% | 19.3% | 3.4% | -11.1% | 18.9% |
| **L** |  |  |  | -2.5% | -14.6% | 21.1% | -4.3% | -17.4% | 19.6% | -5.0% | -18.4% | 19.0% | -0.8% | -12.0% | 22.6% | -3.4% | -14.4% | 19.9% | -1.4% | -19.0% | 23.1% | -1.2% | -16.3% | 22.9% |
| **M** | 2.5% | -21.1% | 14.6% |  |  |  | -1.8% | -11.7% | 7.5% | -2.5% | -12.9% | 7.0% | 1.7% | -5.5% | 9.6% | -0.9% | -7.7% | 6.8% | 1.1% | -14.3% | 11.9% | 1.3% | -11.2% | 11.2% |
| **N** | 4.3% | -19.6% | 17.4% | 1.8% | -7.5% | 11.7% |  |  |  | -0.7% | -11.7% | 10.1% | 3.5% | -4.5% | 12.8% | 0.9% | -6.8% | 10.1% | 2.9% | -12.9% | 14.8% | 3.1% | -9.9% | 14.2% |
| **O** | 5.0% | -19.0% | 18.4% | 2.5% | -7.0% | 12.9% | 0.7% | -10.1% | 11.7% |  |  |  | 4.2% | -4.1% | 14.0% | 1.6% | -6.4% | 11.2% | 3.6% | -12.3% | 15.9% | 3.8% | -9.4% | 15.3% |
| **P** | 0.8% | -22.6% | 12.0% | -1.7% | -9.6% | 5.5% | -3.5% | -12.8% | 4.5% | -4.2% | -14.0% | 4.1% |  |  |  | -2.6% | -8.5% | 3.5% | -0.6% | -15.6% | 9.1% | -0.4% | -12.4% | 8.3% |
| **Q** | 3.4% | -19.9% | 14.4% | 0.9% | -6.8% | 7.7% | -0.9% | -10.1% | 6.8% | -1.6% | -11.2% | 6.4% | 2.6% | -3.5% | 8.5% |  |  |  | 2.0% | -12.9% | 11.5% | 2.2% | -9.7% | 10.6% |
| **R** | 1.4% | -23.1% | 19.0% | -1.1% | -11.9% | 14.3% | -2.9% | -14.8% | 12.9% | -3.6% | -15.9% | 12.3% | 0.6% | -9.1% | 15.6% | -2.0% | -11.5% | 12.9% |  |  |  | 0.2% | -13.9% | 16.4% |
| **Others** | 1.2% | -22.9% | 16.3% | -1.3% | -11.2% | 11.2% | -3.1% | -14.2% | 9.9% | -3.8% | -15.3% | 9.4% | 0.4% | -8.3% | 12.4% | -2.2% | -10.6% | 9.7% | -0.2% | -16.4% | 13.9% |  |  |  |

SIC: Standard Industry Classification

## Supplementary material 9: Estimated prevalence of OSA by occupations in England

Table S11. Estimated prevalence of OSA by occupations in England in 2019 (age 40-64)

| **SOC2010** | **Estimates** | **Lower CI** | **Upper CI** |
| --- | --- | --- | --- |
| (11) Corporate Management | 18.1% | 11.7% | 27.1% |
| (12) Other Management | 17.9% | 9.8% | 30.5% |
| (21) Science and Tech Professionals | 18.4% | 11.3% | 28.4% |
| (22) Health Professionals | 16.0% | 9.2% | 26.4% |
| (23) Teaching Professionals | 15.8% | 9.4% | 25.3% |
| (24) Business and Media Pros | 16.1% | 9.4% | 26.3% |
| (31) Tech Associate Pros | 15.7% | 5.0% | 39.7% |
| (32) Health and Social Care Pros | 21.9% | 9.2% | 43.6% |
| (33) Protective Services | 22.6% | 10.5% | 42.0% |
| (34) Culture and Sports Jobs | 16.2% | 7.9% | 30.3% |
| (35) Business Pros | 18.6% | 12.7% | 26.4% |
| (41) Administrative Jobs | 17.1% | 12.2% | 23.4% |
| (42) Secretarial Jobs | 19.9% | 12.5% | 30.2% |
| (51) Agricultural Trades | 17.7% | 7.4% | 36.6% |
| (52) Metal and Electrical Trades | 17.1% | 9.2% | 29.7% |
| (53) Construction Trades | 21.3% | 12.8% | 33.4% |
| (54) Crafts and Printing | 19.6% | 9.6% | 35.9% |
| (61) Caring Services | 18.3% | 14.0% | 23.6% |
| (62) Leisure and Travel Services | 16.7% | 8.1% | 31.2% |
| (71) Sales Jobs | 18.6% | 12.9% | 26.0% |
| (72) Customer Service | 18.0% | 6.9% | 39.2% |
| (81) Machine Operatives | 20.6% | 13.2% | 30.6% |
| (82) Transport and Drivers | 17.2% | 10.1% | 27.8% |
| (91) Elementary Manual Trades | 14.8% | 5.3% | 35.3% |
| (92) Elementary Administration | 16.4% | 11.7% | 22.6% |
| CI: 95% confidence interval, SOC: Standard Occupation Classification | | | |

Table S12. Pooled differences in estimated prevalence of OSA by occupations in England in 2019 (age40-64)

|  | **Estimates, Lower CI, Upper CI** | | | | | | | | | | | | | | | | | | | | |
| --- | --- | --- | --- | --- | --- | --- | --- | --- | --- | --- | --- | --- | --- | --- | --- | --- | --- | --- | --- | --- | --- |
| **SOC2010** | **11** | | | **12** | | | **21** | | | **22** | | | **23** | | | **24** | | | **31** | | |
| **11** |  |  |  | 0.2% | -13.9% | 12.3% | -0.2% | -12.1% | 11.1% | 2.1% | -10.0% | 13.3% | 2.4% | -9.1% | 13.3% | 2.0% | -10.0% | 13.2% | 2.4% | -22.0% | 16.3% |
| **12** | -0.2% | -12.3% | 13.9% |  |  |  | -0.4% | -13.3% | 13.9% | 1.9% | -11.2% | 16.1% | 2.2% | -10.3% | 16.2% | 1.8% | -11.2% | 16.0% | 2.2% | -22.7% | 18.6% |
| **21** | 0.2% | -11.1% | 12.1% | 0.4% | -13.9% | 13.3% |  |  |  | 2.3% | -10.1% | 14.4% | 2.6% | -9.2% | 14.5% | 2.2% | -10.1% | 14.3% | 2.6% | -22.0% | 17.2% |
| **22** | -2.1% | -13.3% | 10.0% | -1.9% | -16.1% | 11.2% | -2.3% | -14.4% | 10.1% |  |  |  | 0.3% | -11.4% | 12.4% | -0.1% | -12.3% | 12.2% | 0.3% | -24.2% | 15.1% |
| **23** | -2.4% | -13.3% | 9.1% | -2.2% | -16.2% | 10.3% | -2.6% | -14.5% | 9.2% | -0.3% | -12.4% | 11.4% |  |  |  | -0.4% | -12.4% | 11.3% | 0.0% | -24.4% | 14.3% |
| **24** | -2.0% | -13.2% | 10.0% | -1.8% | -16.0% | 11.2% | -2.2% | -14.3% | 10.1% | 0.1% | -12.2% | 12.3% | 0.4% | -11.3% | 12.4% |  |  |  | 0.4% | -24.1% | 15.1% |
| **31** | -2.4% | -16.3% | 22.0% | -2.2% | -18.6% | 22.7% | -2.6% | -17.2% | 22.0% | -0.3% | -15.1% | 24.2% | 0.0% | -14.3% | 24.4% | -0.4% | -15.1% | 24.1% |  |  |  |
| **32** | 3.7% | -11.7% | 26.1% | 3.9% | -13.8% | 26.8% | 3.5% | -12.5% | 26.1% | 5.9% | -10.4% | 28.3% | 6.1% | -9.6% | 28.5% | 5.7% | -10.4% | 28.2% | 6.1% | -20.6% | 30.1% |
| **33** | 4.5% | -10.5% | 24.6% | 4.7% | -12.6% | 25.4% | 4.2% | -11.3% | 24.5% | 6.6% | -9.2% | 26.8% | 6.8% | -8.4% | 26.9% | 6.5% | -9.2% | 26.7% | 6.9% | -19.6% | 28.7% |
| **34** | -2.0% | -14.1% | 13.4% | -1.8% | -16.7% | 14.4% | -2.2% | -15.1% | 13.5% | 0.2% | -13.0% | 15.7% | 0.4% | -12.1% | 15.8% | 0.1% | -13.0% | 15.6% | 0.5% | -24.5% | 18.0% |
| **35** | 0.5% | -10.2% | 10.6% | 0.7% | -13.1% | 11.9% | 0.2% | -11.3% | 10.7% | 2.6% | -9.3% | 12.9% | 2.9% | -8.3% | 12.9% | 2.5% | -9.3% | 12.7% | 2.9% | -21.4% | 16.0% |
| **41** | -1.1% | -11.2% | 7.9% | -0.9% | -14.3% | 9.4% | -1.3% | -12.4% | 8.1% | 1.1% | -10.3% | 10.3% | 1.3% | -9.3% | 10.3% | 0.9% | -10.3% | 10.2% | 1.3% | -22.7% | 13.7% |
| **42** | 1.7% | -9.8% | 13.8% | 2.0% | -12.6% | 15.0% | 1.5% | -10.9% | 13.9% | 3.9% | -8.8% | 16.1% | 4.1% | -7.9% | 16.2% | 3.8% | -8.8% | 16.0% | 4.2% | -20.6% | 18.9% |
| **51** | -0.5% | -14.0% | 19.3% | -0.2% | -16.4% | 20.1% | -0.7% | -14.9% | 19.2% | 1.7% | -12.8% | 21.5% | 1.9% | -12.0% | 21.6% | 1.6% | -12.8% | 21.3% | 2.0% | -23.7% | 23.4% |
| **52** | -1.0% | -13.0% | 13.1% | -0.8% | -15.6% | 14.1% | -1.3% | -14.0% | 13.1% | 1.1% | -11.9% | 15.4% | 1.4% | -11.0% | 15.4% | 1.0% | -11.9% | 15.2% | 1.4% | -23.5% | 17.8% |
| **53** | 3.2% | -9.2% | 16.8% | 3.4% | -11.7% | 17.9% | 2.9% | -10.2% | 16.9% | 5.3% | -8.1% | 19.1% | 5.6% | -7.2% | 19.2% | 5.2% | -8.1% | 19.0% | 5.6% | -19.5% | 21.6% |
| **54** | 1.4% | -11.9% | 18.8% | 1.6% | -14.3% | 19.7% | 1.2% | -12.9% | 18.8% | 3.6% | -10.8% | 21.1% | 3.8% | -9.9% | 21.2% | 3.4% | -10.8% | 20.9% | 3.8% | -21.8% | 23.2% |
| **61** | 0.2% | -9.7% | 8.5% | 0.4% | -12.8% | 10.0% | -0.1% | -10.9% | 8.7% | 2.3% | -8.9% | 10.9% | 2.6% | -7.9% | 10.8% | 2.2% | -8.9% | 10.7% | 2.6% | -21.4% | 14.4% |
| **62** | -1.4% | -13.8% | 14.3% | -1.2% | -16.4% | 15.3% | -1.7% | -14.8% | 14.4% | 0.7% | -12.7% | 16.6% | 0.9% | -11.8% | 16.7% | 0.6% | -12.7% | 16.5% | 1.0% | -24.1% | 18.9% |
| **71** | 0.5% | -10.1% | 10.3% | 0.7% | -13.0% | 11.6% | 0.2% | -11.2% | 10.4% | 2.6% | -9.2% | 12.6% | 2.9% | -8.2% | 12.6% | 2.5% | -9.2% | 12.5% | 2.9% | -21.4% | 15.8% |
| **72** | -0.2% | -14.3% | 21.7% | 0.1% | -16.6% | 22.4% | -0.4% | -15.2% | 21.6% | 2.0% | -13.1% | 23.9% | 2.2% | -12.3% | 24.0% | 1.9% | -13.1% | 23.8% | 2.3% | -23.8% | 25.7% |
| **81** | 2.4% | -9.2% | 14.3% | 2.6% | -11.9% | 15.5% | 2.2% | -10.2% | 14.4% | 4.5% | -8.1% | 16.6% | 4.8% | -7.2% | 16.7% | 4.4% | -8.1% | 16.5% | 4.8% | -19.9% | 19.4% |
| **82** | -0.9% | -12.3% | 11.5% | -0.7% | -15.1% | 12.6% | -1.2% | -13.4% | 11.5% | 1.2% | -11.3% | 13.7% | 1.5% | -10.4% | 13.8% | 1.1% | -11.3% | 13.6% | 1.5% | -23.2% | 16.5% |
| **91** | -3.3% | -16.4% | 18.0% | -3.1% | -18.8% | 18.7% | -3.5% | -17.3% | 17.9% | -1.2% | -15.2% | 20.2% | -0.9% | -14.4% | 20.3% | -1.3% | -15.2% | 20.1% | -0.9% | -26.3% | 22.0% |
| **92** | -1.7% | -11.8% | 7.2% | -1.5% | -14.9% | 8.7% | -2.0% | -13.0% | 7.4% | 0.4% | -10.9% | 9.6% | 0.7% | -9.9% | 9.5% | 0.3% | -10.9% | 9.4% | 0.7% | -23.4% | 13.0% |
|  |  |  |  |  |  |  |  |  |  |  |  |  |  |  |  |  |  |  |  |  |  |
|  | **Estimates, Lower CI, Upper CI** | | | | | | | | | | | | | | | | | | | | |
| **SOC2010** | **32** | | | **33** | | | **34** | | | **35** | | | **41** | | | **42** | | | **51** | | |
| **11** | -3.7% | -26.1% | 11.7% | -4.5% | -24.6% | 10.5% | 2.0% | -13.4% | 14.1% | -0.5% | -10.6% | 10.2% | 1.1% | -7.9% | 11.2% | -1.7% | -13.8% | 9.8% | 0.5% | -19.3% | 14.0% |
| **12** | -3.9% | -26.8% | 13.8% | -4.7% | -25.4% | 12.6% | 1.8% | -14.4% | 16.7% | -0.7% | -11.9% | 13.1% | 0.9% | -9.4% | 14.3% | -2.0% | -15.0% | 12.6% | 0.2% | -20.1% | 16.4% |
| **21** | -3.5% | -26.1% | 12.5% | -4.2% | -24.5% | 11.3% | 2.2% | -13.5% | 15.1% | -0.2% | -10.7% | 11.3% | 1.3% | -8.1% | 12.4% | -1.5% | -13.9% | 10.9% | 0.7% | -19.2% | 14.9% |
| **22** | -5.9% | -28.3% | 10.4% | -6.6% | -26.8% | 9.2% | -0.2% | -15.7% | 13.0% | -2.6% | -12.9% | 9.3% | -1.1% | -10.3% | 10.3% | -3.9% | -16.1% | 8.8% | -1.7% | -21.5% | 12.8% |
| **23** | -6.1% | -28.5% | 9.6% | -6.8% | -26.9% | 8.4% | -0.4% | -15.8% | 12.1% | -2.9% | -12.9% | 8.3% | -1.3% | -10.3% | 9.3% | -4.1% | -16.2% | 7.9% | -1.9% | -21.6% | 12.0% |
| **24** | -5.7% | -28.2% | 10.4% | -6.5% | -26.7% | 9.2% | -0.1% | -15.6% | 13.0% | -2.5% | -12.7% | 9.3% | -0.9% | -10.2% | 10.3% | -3.8% | -16.0% | 8.8% | -1.6% | -21.3% | 12.8% |
| **31** | -6.1% | -30.1% | 20.6% | -6.9% | -28.7% | 19.6% | -0.5% | -18.0% | 24.5% | -2.9% | -16.0% | 21.4% | -1.3% | -13.7% | 22.7% | -4.2% | -18.9% | 20.6% | -2.0% | -23.4% | 23.7% |
| **32** |  |  |  | -0.7% | -23.6% | 23.8% | 5.7% | -13.1% | 28.7% | 3.3% | -11.5% | 25.5% | 4.8% | -9.3% | 26.8% | 2.0% | -14.2% | 24.7% | 4.2% | -18.3% | 27.9% |
| **33** | 0.7% | -23.8% | 23.6% |  |  |  | 6.4% | -12.0% | 27.2% | 4.0% | -10.3% | 23.9% | 5.5% | -8.0% | 25.2% | 2.7% | -13.0% | 23.2% | 4.9% | -17.2% | 26.5% |
| **34** | -5.7% | -28.7% | 13.1% | -6.4% | -27.2% | 12.0% |  |  |  | -2.4% | -13.7% | 12.7% | -0.9% | -11.3% | 13.9% | -3.7% | -16.8% | 12.1% | -1.5% | -21.9% | 15.8% |
| **35** | -3.3% | -25.5% | 11.5% | -4.0% | -23.9% | 10.3% | 2.4% | -12.7% | 13.7% |  |  |  | 1.5% | -7.1% | 10.7% | -1.3% | -13.1% | 9.4% | 0.9% | -18.6% | 13.7% |
| **41** | -4.8% | -26.8% | 9.3% | -5.5% | -25.2% | 8.0% | 0.9% | -13.9% | 11.3% | -1.5% | -10.7% | 7.1% |  |  |  | -2.8% | -14.1% | 6.9% | -0.6% | -19.8% | 11.4% |
| **42** | -2.0% | -24.7% | 14.2% | -2.7% | -23.2% | 13.0% | 3.7% | -12.1% | 16.8% | 1.3% | -9.4% | 13.1% | 2.8% | -6.9% | 14.1% |  |  |  | 2.2% | -17.8% | 16.6% |
| **51** | -4.2% | -27.9% | 18.3% | -4.9% | -26.5% | 17.2% | 1.5% | -15.8% | 21.9% | -0.9% | -13.7% | 18.6% | 0.6% | -11.4% | 19.8% | -2.2% | -16.6% | 17.8% |  |  |  |
| **52** | -4.8% | -27.6% | 13.0% | -5.5% | -26.1% | 11.9% | 0.9% | -15.1% | 16.0% | -1.5% | -12.6% | 12.4% | 0.0% | -10.1% | 13.5% | -2.8% | -15.7% | 11.8% | -0.6% | -20.8% | 15.6% |
| **53** | -0.6% | -23.6% | 16.8% | -1.3% | -22.2% | 15.7% | 5.1% | -11.3% | 19.7% | 2.7% | -8.8% | 16.1% | 4.2% | -6.4% | 17.2% | 1.4% | -11.9% | 15.5% | 3.6% | -16.9% | 19.4% |
| **54** | -2.3% | -25.9% | 18.2% | -3.0% | -24.5% | 17.1% | 3.4% | -13.8% | 21.5% | 1.0% | -11.6% | 18.1% | 2.5% | -9.3% | 19.4% | -0.3% | -14.6% | 17.4% | 1.9% | -19.2% | 21.0% |
| **61** | -3.6% | -25.4% | 10.1% | -4.3% | -23.8% | 8.8% | 2.1% | -12.5% | 11.9% | -0.3% | -9.2% | 7.6% | 1.2% | -6.4% | 8.4% | -1.6% | -12.7% | 7.5% | 0.6% | -18.5% | 12.1% |
| **62** | -5.2% | -28.2% | 13.9% | -5.9% | -26.8% | 12.8% | 0.5% | -15.9% | 17.1% | -1.9% | -13.4% | 13.6% | -0.4% | -11.0% | 14.8% | -3.2% | -16.5% | 13.0% | -1.0% | -21.5% | 16.6% |
| **71** | -3.3% | -25.4% | 11.3% | -4.0% | -23.9% | 10.1% | 2.4% | -12.7% | 13.5% | 0.0% | -9.6% | 9.4% | 1.5% | -6.9% | 10.4% | -1.3% | -13.0% | 9.2% | 0.9% | -18.5% | 13.5% |
| **72** | -3.9% | -28.0% | 20.5% | -4.6% | -26.6% | 19.4% | 1.8% | -16.0% | 24.2% | -0.6% | -14.0% | 21.0% | 0.9% | -11.7% | 22.3% | -1.9% | -16.9% | 20.2% | 0.3% | -21.3% | 23.5% |
| **81** | -1.3% | -24.0% | 14.7% | -2.0% | -22.5% | 13.5% | 4.4% | -11.4% | 17.3% | 1.9% | -8.8% | 13.6% | 3.5% | -6.2% | 14.6% | 0.7% | -11.9% | 13.1% | 2.9% | -17.2% | 17.2% |
| **82** | -4.7% | -27.2% | 11.8% | -5.4% | -25.7% | 10.6% | 1.0% | -14.7% | 14.4% | -1.4% | -11.9% | 10.7% | 0.1% | -9.4% | 11.8% | -2.7% | -15.1% | 10.2% | -0.5% | -20.4% | 14.2% |
| **91** | -7.1% | -30.5% | 16.8% | -7.8% | -29.1% | 15.8% | -1.4% | -18.3% | 20.5% | -3.8% | -16.1% | 17.3% | -2.3% | -13.7% | 18.6% | -5.1% | -19.0% | 16.5% | -2.9% | -23.8% | 19.8% |
| **92** | -5.5% | -27.4% | 8.6% | -6.2% | -25.8% | 7.3% | 0.2% | -14.5% | 10.5% | -2.2% | -11.3% | 6.3% | -0.7% | -8.5% | 7.2% | -3.5% | -14.7% | 6.2% | -1.3% | -20.5% | 10.6% |
|  |  |  |  |  |  |  |  |  |  |  |  |  |  |  |  |  |  |  |  |  |  |
|  |  |  |  |  |  |  |  |  |  |  |  |  |  |  |  |  |  |  |  |  |  |
|  | **Estimates, Lower CI, Upper CI** | | | | | | | | | | | | | | | | | | | | |
| **SOC2010** | **52** | | | **53** | | | **54** | | | **61** | | | **62** | | | **71** | | | **72** | | |
| **11** | 1.0% | -13.1% | 13.0% | -3.2% | -16.8% | 9.2% | -1.4% | -18.8% | 11.9% | -0.2% | -8.5% | 9.7% | 1.4% | -14.3% | 13.8% | -0.5% | -10.3% | 10.1% | 0.2% | -21.7% | 14.3% |
| **12** | 0.8% | -14.1% | 15.6% | -3.4% | -17.9% | 11.7% | -1.6% | -19.7% | 14.3% | -0.4% | -10.0% | 12.8% | 1.2% | -15.3% | 16.4% | -0.7% | -11.6% | 13.0% | -0.1% | -22.4% | 16.6% |
| **21** | 1.3% | -13.1% | 14.0% | -2.9% | -16.9% | 10.2% | -1.2% | -18.8% | 12.9% | 0.1% | -8.7% | 10.9% | 1.7% | -14.4% | 14.8% | -0.2% | -10.4% | 11.2% | 0.4% | -21.6% | 15.2% |
| **22** | -1.1% | -15.4% | 11.9% | -5.3% | -19.1% | 8.1% | -3.6% | -21.1% | 10.8% | -2.3% | -10.9% | 8.9% | -0.7% | -16.6% | 12.7% | -2.6% | -12.6% | 9.2% | -2.0% | -23.9% | 13.1% |
| **23** | -1.4% | -15.4% | 11.0% | -5.6% | -19.2% | 7.2% | -3.8% | -21.2% | 9.9% | -2.6% | -10.8% | 7.9% | -0.9% | -16.7% | 11.8% | -2.9% | -12.6% | 8.2% | -2.2% | -24.0% | 12.3% |
| **24** | -1.0% | -15.2% | 11.9% | -5.2% | -19.0% | 8.1% | -3.4% | -20.9% | 10.8% | -2.2% | -10.7% | 8.9% | -0.6% | -16.5% | 12.7% | -2.5% | -12.5% | 9.2% | -1.9% | -23.8% | 13.1% |
| **31** | -1.4% | -17.8% | 23.5% | -5.6% | -21.6% | 19.5% | -3.8% | -23.2% | 21.8% | -2.6% | -14.4% | 21.4% | -1.0% | -18.9% | 24.1% | -2.9% | -15.8% | 21.4% | -2.3% | -25.7% | 23.8% |
| **32** | 4.8% | -13.0% | 27.6% | 0.6% | -16.8% | 23.6% | 2.3% | -18.2% | 25.9% | 3.6% | -10.1% | 25.4% | 5.2% | -13.9% | 28.2% | 3.3% | -11.3% | 25.4% | 3.9% | -20.5% | 28.0% |
| **33** | 5.5% | -11.9% | 26.1% | 1.3% | -15.7% | 22.2% | 3.0% | -17.1% | 24.5% | 4.3% | -8.8% | 23.8% | 5.9% | -12.8% | 26.8% | 4.0% | -10.1% | 23.9% | 4.6% | -19.4% | 26.6% |
| **34** | -0.9% | -16.0% | 15.1% | -5.1% | -19.7% | 11.3% | -3.4% | -21.5% | 13.8% | -2.1% | -11.9% | 12.5% | -0.5% | -17.1% | 15.9% | -2.4% | -13.5% | 12.7% | -1.8% | -24.2% | 16.0% |
| **35** | 1.5% | -12.4% | 12.6% | -2.7% | -16.1% | 8.8% | -1.0% | -18.1% | 11.6% | 0.3% | -7.6% | 9.2% | 1.9% | -13.6% | 13.4% | 0.0% | -9.4% | 9.6% | 0.6% | -21.0% | 14.0% |
| **41** | 0.0% | -13.5% | 10.1% | -4.2% | -17.2% | 6.4% | -2.5% | -19.4% | 9.3% | -1.2% | -8.4% | 6.4% | 0.4% | -14.8% | 11.0% | -1.5% | -10.4% | 6.9% | -0.9% | -22.3% | 11.7% |
| **42** | 2.8% | -11.8% | 15.7% | -1.4% | -15.5% | 11.9% | 0.3% | -17.4% | 14.6% | 1.6% | -7.5% | 12.7% | 3.2% | -13.0% | 16.5% | 1.3% | -9.2% | 13.0% | 1.9% | -20.2% | 16.9% |
| **51** | 0.6% | -15.6% | 20.8% | -3.6% | -19.4% | 16.9% | -1.9% | -21.0% | 19.2% | -0.6% | -12.1% | 18.5% | 1.0% | -16.6% | 21.5% | -0.9% | -13.5% | 18.5% | -0.3% | -23.5% | 21.3% |
| **52** |  |  |  | -4.2% | -18.6% | 11.0% | -2.5% | -20.5% | 13.6% | -1.2% | -10.7% | 12.1% | 0.4% | -16.0% | 15.6% | -1.5% | -12.3% | 12.3% | -0.9% | -23.2% | 15.8% |
| **53** | 4.2% | -11.0% | 18.6% |  |  |  | 1.7% | -16.5% | 17.3% | 3.0% | -7.0% | 15.8% | 4.6% | -12.1% | 19.4% | 2.7% | -8.6% | 16.0% | 3.3% | -19.2% | 19.6% |
| **54** | 2.5% | -13.6% | 20.5% | -1.7% | -17.3% | 16.5% |  |  |  | 1.3% | -10.0% | 18.0% | 2.9% | -14.6% | 21.1% | 1.0% | -11.4% | 18.1% | 1.6% | -21.5% | 21.1% |
| **61** | 1.2% | -12.1% | 10.7% | -3.0% | -15.8% | 7.0% | -1.3% | -18.0% | 10.0% |  |  |  | 1.6% | -13.4% | 11.6% | -0.3% | -8.8% | 7.4% | 0.3% | -21.0% | 12.5% |
| **62** | -0.4% | -15.6% | 16.0% | -4.6% | -19.4% | 12.1% | -2.9% | -21.1% | 14.6% | -1.6% | -11.6% | 13.4% |  |  |  | -1.9% | -13.2% | 13.6% | -1.3% | -23.8% | 16.8% |
| **71** | 1.5% | -12.3% | 12.3% | -2.7% | -16.0% | 8.6% | -1.0% | -18.1% | 11.4% | 0.3% | -7.4% | 8.8% | 1.9% | -13.6% | 13.2% |  |  |  | 0.6% | -21.0% | 13.8% |
| **72** | 0.9% | -15.8% | 23.2% | -3.3% | -19.6% | 19.2% | -1.6% | -21.1% | 21.5% | -0.3% | -12.5% | 21.0% | 1.3% | -16.8% | 23.8% | -0.6% | -13.8% | 21.0% |  |  |  |
| **81** | 3.4% | -11.1% | 16.2% | -0.8% | -14.9% | 12.4% | 1.0% | -16.8% | 15.1% | 2.2% | -6.8% | 13.1% | 3.9% | -12.3% | 17.0% | 2.0% | -8.5% | 13.4% | 2.6% | -19.5% | 17.4% |
| **82** | 0.1% | -14.3% | 13.3% | -4.1% | -18.1% | 9.5% | -2.4% | -20.0% | 12.1% | -1.1% | -10.0% | 10.3% | 0.5% | -15.5% | 14.1% | -1.4% | -11.6% | 10.6% | -0.8% | -22.8% | 14.4% |
| **91** | -2.3% | -18.1% | 19.5% | -6.5% | -21.8% | 15.5% | -4.8% | -23.5% | 17.8% | -3.5% | -14.4% | 17.2% | -1.9% | -19.1% | 20.1% | -3.8% | -15.8% | 17.3% | -3.2% | -26.1% | 19.9% |
| **92** | -0.7% | -14.1% | 9.4% | -4.9% | -17.8% | 5.6% | -3.2% | -20.0% | 8.6% | -1.9% | -9.0% | 5.6% | -0.3% | -15.4% | 10.3% | -2.2% | -11.0% | 6.2% | -1.6% | -23.0% | 11.0% |
|  |  |  |  |  |  |  |  |  |  |  |  |  |  |  |  |  |  |  |  |  |  |
|  | **Estimates, Lower CI, Upper CI** | | | | | | | | | | | |  |  |  |  |  |  |  |  |  |
| **SOC2010** | **81** | | | **82** | | | **91** | | | **92** | | |  |  |  |  |  |  |  |  |  |
| **11** | -2.4% | -14.3% | 9.2% | 0.9% | -11.5% | 12.3% | 3.3% | -18.0% | 16.4% | 1.7% | -7.2% | 11.8% |  |  |  |  |  |  |  |  |  |
| **12** | -2.6% | -15.5% | 11.9% | 0.7% | -12.6% | 15.1% | 3.1% | -18.7% | 18.8% | 1.5% | -8.7% | 14.9% |  |  |  |  |  |  |  |  |  |
| **21** | -2.2% | -14.4% | 10.2% | 1.2% | -11.5% | 13.4% | 3.5% | -17.9% | 17.3% | 2.0% | -7.4% | 13.0% |  |  |  |  |  |  |  |  |  |
| **22** | -4.5% | -16.6% | 8.1% | -1.2% | -13.7% | 11.3% | 1.2% | -20.2% | 15.2% | -0.4% | -9.6% | 10.9% |  |  |  |  |  |  |  |  |  |
| **23** | -4.8% | -16.7% | 7.2% | -1.5% | -13.8% | 10.4% | 0.9% | -20.3% | 14.4% | -0.7% | -9.5% | 9.9% |  |  |  |  |  |  |  |  |  |
| **24** | -4.4% | -16.5% | 8.1% | -1.1% | -13.6% | 11.3% | 1.3% | -20.1% | 15.2% | -0.3% | -9.4% | 10.9% |  |  |  |  |  |  |  |  |  |
| **31** | -4.8% | -19.4% | 19.9% | -1.5% | -16.5% | 23.2% | 0.9% | -22.0% | 26.3% | -0.7% | -13.0% | 23.4% |  |  |  |  |  |  |  |  |  |
| **32** | 1.3% | -14.7% | 24.0% | 4.7% | -11.8% | 27.2% | 7.1% | -16.8% | 30.5% | 5.5% | -8.6% | 27.4% |  |  |  |  |  |  |  |  |  |
| **33** | 2.0% | -13.5% | 22.5% | 5.4% | -10.6% | 25.7% | 7.8% | -15.8% | 29.1% | 6.2% | -7.3% | 25.8% |  |  |  |  |  |  |  |  |  |
| **34** | -4.4% | -17.3% | 11.4% | -1.0% | -14.4% | 14.7% | 1.4% | -20.5% | 18.3% | -0.2% | -10.5% | 14.5% |  |  |  |  |  |  |  |  |  |
| **35** | -1.9% | -13.6% | 8.8% | 1.4% | -10.7% | 11.9% | 3.8% | -17.3% | 16.1% | 2.2% | -6.3% | 11.3% |  |  |  |  |  |  |  |  |  |
| **41** | -3.5% | -14.6% | 6.2% | -0.1% | -11.8% | 9.4% | 2.3% | -18.6% | 13.7% | 0.7% | -7.2% | 8.5% |  |  |  |  |  |  |  |  |  |
| **42** | -0.7% | -13.1% | 11.9% | 2.7% | -10.2% | 15.1% | 5.1% | -16.5% | 19.0% | 3.5% | -6.2% | 14.7% |  |  |  |  |  |  |  |  |  |
| **51** | -2.9% | -17.2% | 17.2% | 0.5% | -14.2% | 20.4% | 2.9% | -19.8% | 23.8% | 1.3% | -10.6% | 20.5% |  |  |  |  |  |  |  |  |  |
| **52** | -3.4% | -16.2% | 11.1% | -0.1% | -13.3% | 14.3% | 2.3% | -19.5% | 18.1% | 0.7% | -9.4% | 14.1% |  |  |  |  |  |  |  |  |  |
| **53** | 0.8% | -12.4% | 14.9% | 4.1% | -9.5% | 18.1% | 6.5% | -15.5% | 21.8% | 4.9% | -5.6% | 17.8% |  |  |  |  |  |  |  |  |  |
| **54** | -1.0% | -15.1% | 16.8% | 2.4% | -12.1% | 20.0% | 4.8% | -17.8% | 23.5% | 3.2% | -8.6% | 20.0% |  |  |  |  |  |  |  |  |  |
| **61** | -2.2% | -13.1% | 6.8% | 1.1% | -10.3% | 10.0% | 3.5% | -17.2% | 14.4% | 1.9% | -5.6% | 9.0% |  |  |  |  |  |  |  |  |  |
| **62** | -3.9% | -17.0% | 12.3% | -0.5% | -14.1% | 15.5% | 1.9% | -20.1% | 19.1% | 0.3% | -10.3% | 15.4% |  |  |  |  |  |  |  |  |  |
| **71** | -2.0% | -13.4% | 8.5% | 1.4% | -10.6% | 11.6% | 3.8% | -17.3% | 15.8% | 2.2% | -6.2% | 11.0% |  |  |  |  |  |  |  |  |  |
| **72** | -2.6% | -17.4% | 19.5% | 0.8% | -14.4% | 22.8% | 3.2% | -19.9% | 26.1% | 1.6% | -11.0% | 23.0% |  |  |  |  |  |  |  |  |  |
| **81** |  |  |  | 3.3% | -9.5% | 15.6% | 5.7% | -15.8% | 19.5% | 4.1% | -5.5% | 15.2% |  |  |  |  |  |  |  |  |  |
| **82** | -3.3% | -15.6% | 9.5% |  |  |  | 2.4% | -19.1% | 16.6% | 0.8% | -8.6% | 12.4% |  |  |  |  |  |  |  |  |  |
| **91** | -5.7% | -19.5% | 15.8% | -2.4% | -16.6% | 19.1% |  |  |  | -1.6% | -12.9% | 19.2% |  |  |  |  |  |  |  |  |  |
| **92** | -4.1% | -15.2% | 5.5% | -0.8% | -12.4% | 8.6% | 1.6% | -19.2% | 12.9% |  |  |  |  |  |  |  |  |  |  |  |  |

SOC: Standard Occupational Classification

## References

1. Szymański FM, Puchalski B, Filipiak KJ. Obstructive sleep apnea, atrial fibrillation, and erectile dysfunction: are they only coexisting conditions or a new clinical syndrome? The concept of the OSAFED syndrome. Pol Arch Med Wewn. 2013; 123 (12): 701-707.

2. Jordan AS, McSharry DG, Malhotra A. Adult obstructive sleep apnoea. The Lancet. 2014; 383 (9918): 736-747.

3. Woods CE, Usher K, Maguire GP. Obstructive sleep apnoea in adult indigenous populations in high-income countries: an integrative review. Sleep Breath. 2015; 19 (1): 45-53.

4. Cowie MR. Sleep apnea: State of the art. Trends in Cardiovascular Medicine. 2017; 27 (4): 280-289.

5. Papadopoulos D, Kikemeni A, Skourti A, Amfilochiou A. The influence of socio-economic status on the severity of obstructive sleep apnea: a cross-sectional observational study. Sleep Sci. 2018; 11 (2): 92-98.

6. Lee JJ, Sundar KM. Evaluation and Management of Adults with Obstructive Sleep Apnea Syndrome. Lung. 2021; 199 (2): 87-101.

7. Mitra AK, Bhuiyan AR, Jones EA. Association and Risk Factors for Obstructive Sleep Apnea and Cardiovascular Diseases: A Systematic Review. Diseases. 2021; 9 (4).

8. Erridge S, Moussa O, McIntyre C, et al. Obstructive Sleep Apnea in Obese Patients: a UK Population Analysis. Obesity Surgery. 2021; 31 (5): 1986-1993.

9. Sutherland K, Lee RW, Cistulli PA. Obesity and craniofacial structure as risk factors for obstructive sleep apnoea: impact of ethnicity. Respirology. 2012; 17 (2): 213-222.

10. Dudley KA, Patel SR. Disparities and genetic risk factors in obstructive sleep apnea. Sleep Med. 2016; 18: 96-102.

11. Mirrakhimov AE. Obstructive sleep apnea and kidney disease: is there any direct link? Sleep Breath. 2012; 16 (4): 1009-1016.

12. Mirrakhimov AE. Supine fluid redistribution: should we consider this as an important risk factor for obstructive sleep apnea? Sleep Breath. 2013; 17 (2): 511-523.

13. Jhamb M, Unruh M. Bidirectional relationship of hypertension with obstructive sleep apnea. Curr Opin Pulm Med. 2014; 20 (6): 558-564.

14. He L, Wang B, Lang WY, et al. Genetically-reduced serum ACE activity might be a causal risk factor for obstructive sleep apnea syndrome: A meta-analysis. Sci Rep. 2015; 5: 15267.

15. Yuan F, Zhang S, Liu X, Liu Y. Correlation between obstructive sleep apnea hypopnea syndrome and hypertension: a systematic review and meta-analysis. Ann Palliat Med. 2021; 10 (12): 12251-12261.

16. Manin G, Pons A, Baltzinger P, et al. Obstructive sleep apnoea in people with Type 1 diabetes: prevalence and association with micro- and macrovascular complications. Diabet Med. 2015; 32 (1): 90-96.

17. Song SO, He K, Narla RR, Kang HG, Ryu HU, Boyko EJ. Metabolic Consequences of Obstructive Sleep Apnea Especially Pertaining to Diabetes Mellitus and Insulin Sensitivity. Diabetes Metab J. 2019; 43 (2): 144-155.

18. National Sleep Research Resource. Sleep Heart Health Study. In. National Sleep Research Resource [dataset] 2014. <https://doi.org/10.25822/ghy8-ks59>

19. NatCen Social Research UCL, Department of Epidemiology and Public Health. Health Survey for England, 2019 In. UK Data Service [dataset] 2021. <https://doi.org/10.5255/UKDA-SN-8860-1>

20. National Sleep Research Resource. Wisconsin Sleep Cohort. In. National Sleep Research Resource [dataset] 2020. <https://doi.org/10.25822/js0k-yh52>.

21. National Sleep Research Resource Sleep Heart Health Study. Sleep Heart Health Study Variables. <https://sleepdata.org/datasets/shhs/variables>. Accessed 15 July, 2022.

22. Ferguson KD, McCann M, Katikireddi SV, et al. Corrigendum to: Evidence synthesis for constructing directed acyclic graphs (ESC-DAGs): a novel and systematic method for building directed acyclic graphs. Int J Epidemiol. 2019; 49 (1): 353-353.

23. Shamela Pepper-Grainger. Age and date of birth harmonised standard. <https://gss.civilservice.gov.uk/policy-store/age-and-date-of-birth/>. Accessed 13 July, 2022.

24. Rugge B, Balshem H, Sehgal R, Relevo R, Gorman P, Helfand M. AHRQ Comparative Effectiveness Reviews. In: Screening and Treatment of Subclinical Hypothyroidism or Hyperthyroidism. Rockville (MD): Agency for Healthcare Research and Quality (US); 2011.

25. NatCen Social Research and UCL. *Health Survey for England 2019 Methods.* 2020.

26. NatCen Social Research UCL, Department of Epidemiology and Public Health, . Health Survey for England, 2019. In: Service UD, ed2021.

27. Quan SF, Howard BV, Iber C, et al. The Sleep Heart Health Study: design, rationale, and methods. Sleep. 1997; 20 (12): 1077-1085.

28. Young T, Palta M, Dempsey J, Peppard PE, Nieto FJ, Hla KM. Burden of sleep apnea: rationale, design, and major findings of the Wisconsin Sleep Cohort study. Wmj. 2009; 108 (5): 246-249.

29. Eekhout I, van de Wiel MA, Heymans MW. Methods for significance testing of categorical covariates in logistic regression models after multiple imputation: power and applicability analysis. BMC Med Res Methodol. 2017; 17 (1): 129.

30. Heymans M. miceafter: Data and Statistical Analyses after Multiple Imputation. 2022. <https://mwheymans.github.io/miceafter/>.
